# Cryptic indole hydroxylation by a non-canonical terpenoid cyclase parallels bacterial xenobiotic detoxification

**DOI:** 10.1038/ncomms15804

**Published:** 2017-06-15

**Authors:** Susann Kugel, Martin Baunach, Philipp Baer, Mie Ishida-Ito, Srividhya Sundaram, Zhongli Xu, Michael Groll, Christian Hertweck

**Affiliations:** 1 Department of Biomolecular Chemistry, Leibniz Institute for Natural Product Research and Infection Biology (HKI), Beutenbergstr. 11a, 07745 Jena, Germany; 2 Center for Integrated Protein Science Munich (CIPSM), Department Chemie, Technische Universität München, Lichtenbergstr. 4, 85748 Garching, Germany; 3 Natural Product Chemistry, Friedrich Schiller University, 07743 Jena, Germany

## Abstract

Terpenoid natural products comprise a wide range of molecular architectures that typically result from C–C bond formations catalysed by classical type I/II terpene cyclases. However, the molecular diversity of biologically active terpenoids is substantially increased by fully unrelated, non-canonical terpenoid cyclases. Their evolutionary origin has remained enigmatic. Here we report the *in vitro* reconstitution of an unusual flavin-dependent bacterial indoloterpenoid cyclase, XiaF, together with a designated flavoenzyme-reductase (XiaP) that mediates a key step in xiamycin biosynthesis. The crystal structure of XiaF with bound FADH_2_ (at 2.4 Å resolution) and phylogenetic analyses reveal that XiaF is, surprisingly, most closely related to xenobiotic-degrading enzymes. Biotransformation assays show that XiaF is a designated indole hydroxylase that can be used for the production of indigo and indirubin. We unveil a cryptic hydroxylation step that sets the basis for terpenoid cyclization and suggest that the cyclase has evolved from xenobiotics detoxification enzymes.

## Abstract (web summary)

The biosynthesis of xiamycin, an antimicrobial bacterial indolosesquiterpenoid, involves an unusual cyclization cascade. Here, the authors characterise the XiaF enzyme, which resembles xenobiont-degrading enzymes and is responsible for a hidden indole hydroxylation step that triggers the cyclization reaction.

It is a generally accepted model that the vast structural diversity of secondary metabolites has developed to address specific needs of the producing organism. Such natural products may, for example, serve as carrier of information or as chemical armour; they may be used to attract partners, to secure the niche, to protect against various types of stresses and to shape communities^1^,^2^. Natural products are also a valuable source of molecular tools and therapeutics that have been pre-optimized for particular biological targets over millions of years^3^. However, considering the impressive range of structures and functions, one of the remaining big questions is: how has this structural diversity evolved?

Research in the past decades has led to an impressive body of knowledge on biosynthetic pathways, their genetic basis and the biocatalysts involved^4^,^5^,^6^. Typically, there are various levels of diversification in each biosynthetic pathway, such as the choice and assembly of basic building blocks, the processing of various intermediates and various ways to tailor the individual scaffolds^4^,^7^. Typically, enzymes involved in secondary metabolite biosynthesis are rather specific^8^. They are often fine-tuned to direct reactive intermediates into specific reaction channels, and small changes may lead to dramatic changes in the metabolic profile^9^,^10^. This principle is well exemplified in terpene biosynthesis, where designated cyclases orchestrate a series of cation–olefin cyclizations and Wagner–Meerwein rearrangements of intermediates to produce diverse carbocyclic systems^11^,^12^. Beyond these classical type I/II terpene cyclases, there is a surprisingly broad range of non-canonical terpenoid cyclases^13^,^14^.

Various atypical terpenoid cyclizations and diversifications are involved in the pathway leading to the xiamycin family of indolosesquiterpenes (IST) (Fig. 1). These ‘plant-like’ terpenoids are actually produced by bacterial endophytes (*Streptomyces* spp.). Endowed with a range of antiviral^15^,^16^,^17^ and antimicrobial activities^18^,^19^,^20^,^21^,^22^,^23^,^24^ xiamycin and its congeners may aid in protecting the plant host from infections^16^,^20^.

To elucidate the molecular basis of IST diversification, we investigated the xiamycin (*xia*) biosynthetic gene cluster in the endophyte. We found that xiamycin (**3**), the major IST component of the complex^20^, is diversified by means of rare flavoenzyme-catalysed modifications to yield the cycloether oxiamycin (**5**)^18^,^23^, various *N,C*- and *N,N*-fused xiamycin dimers (such as **6a/6b**)^18^,^22^,^23^, and sulfonyl-bridged xiamycin dimers (such as **7**)^19^ (Fig. 1). At the heart of the biosynthetic pathway, however, we discovered an unusual cyclization cascade involving the non-canonical terpenoid cyclases XiaE and XiaF^22^. The first cyclization yields the decalin system of indosespene (**2**), which is further cyclized into the spiro compound sespenine (**4**) or the carbazole ring system of xiamycin (Fig. 1)^13^,^21^,^22^,^25^,^26^.

This non-canonical terpenoid cyclization is mechanistically intriguing and has been the subject of several biosynthetic^21^,^22^,^26^ and synthetic studies^17^,^25^. Through bioinformatics and mutational analysis, we found that XiaF is a flavin-dependent monooxygenase that catalyses the conversion of **2** to the carbazole scaffold of **3** (ref. 22). In an independent study, an orthologue from *Streptomyces* sp. SCSIO 02999 (XiaI, according to these authors’ nomenclature) was found to catalyse this reaction *in vitro*, albeit with unsatisfactory efficacy^21^. Yet, the detailed enzymatic mechanism of this rare cyclization reaction and the evolutionary origin of this extraordinary oxygenase/cyclase have remained elusive.

Here we report the *in vitro* reconstitution of XiaF and a designated flavoenzyme-reductase, which jointly mediate a cryptic indole hydroxylation step that has been proposed to be a key step in non-canonical terpenoid cyclization. By phylogenetic, functional and structural analyses, we also demonstrate the relatedness of this rare terpenoid cyclase with broad-specific xenobiotic-metabolizing enzymes.

## Results

### Reconstitution of two-component terpenoid cyclization

To gain insight into the biochemical function of the indosespene cyclase XiaF, we produced XiaF in *Escherichia coli*. Initially, the *in vitro* characterization of recombinant XiaF was hampered by its poor performance. Substantial biotransformation of **2** (100 μM) was only achieved after extended incubation times (12–24 h) with high concentrations of enzyme (25 μM) and cofactors FAD and NADH (1 and 2 mM). Similar observations were reported for enzyme assays using the XiaF orthologue XiaI from *Streptomyces* sp. SCSIO 02999 (refs 21, 26). However, even under these optimized non-physiological reaction conditions, the catalytic performance of XiaF was unreliable.

To overcome these shortcomings, we investigated the effect of added NAD(P)H:flavin oxidoreductase (flavin reductase) for cofactor recycling. In fact, terpenoid cyclization was efficiently reconstituted *in vitro* using Fre, the flavin reductase from *E. coli* (Supplementary Fig. 2). This finding strongly suggested that XiaF naturally acts in a two-component system. It is conceivable that XiaF variants are orphan oxygenases^27^ that are supported by unrelated flavin reductases derived from the central metabolism. Alternatively, XiaF variants may have a designated reductase partner. Thus, we revisited the *xia* gene locus^22^ and related gene clusters of various other actinomycetes^26^. Genome mining revealed a cryptic gene cluster in *Amycolatopsis nigrescens* CSC17Ta-90 with high similarity to the *xia* gene locus. In contrast to the *xia* gene cluster in the endophytic *Streptomyces* sp. (HKI0576), a putative flavin reductase gene is located next to the *xiaF* orthologue (Fig. 2a). On the basis of this sequence, we screened the genome sequence of *Streptomyces* sp. (HKI0576) and discovered a homologous flavin reductase gene (named *xiaP*) located in the flanking region of the *xia* gene cluster (Fig. 2a; the *xiaP* orthologue from *A. nigrescens* CSC17Ta-90 was named *xiaP′*). The deduced gene products of both *xiaP* variants share homology with other flavin reductase partners of XiaF homologues^28^,^29^,^30^,^31^,^32^,^33^,^34^ (Supplementary Fig. 5).

To test whether XiaP functions as flavin reductase partner of XiaF, we cloned and heterologously expressed *xiaP* from *Streptomyces* sp. (HKI0576) in *E. coli*. Flavin reduction activity assays of XiaP with NAD(P)H and FAD, FMN or riboflavin confirmed its proposed function. They revealed a dependency of XiaP on NADH, whereas NADPH seems to be no suitable cofactor. In contrast, XiaP seems to have no marked preference for any flavin substrate tested (Fig. 3c; for a detailed kinetic analysis of XiaP, see the Supplementary Note 2). When substituting Fre by XiaP in the XiaF enzyme assays, we found that XiaP substantially increases the performance of XiaF (Fig. 3b,I). Since the corresponding genes are found in the same gene locus, XiaP likely acts as the natural flavin reductase for XiaF, although its function may be substituted by other flavin reductases.

Using this robust enzyme assay, we set out to analyse the course of the reaction. In the current biosynthetic model, an oxidative cyclization sequence is proposed in which hydroxylation of **2** is presumed to trigger nucleophilic attack of the *exo*-methylene group followed by dehydratation to yield **3** (refs 20, 21, 22). Furthermore, the immediate product of the XiaF-catalysed reaction has been proposed to be a dihydrocarbazole, which most likely undergoes spontaneous oxidation to give the carbazole **3** (ref. 21). By liquid chromatography high-resolution mass spectrometry (LC-HRMS) monitoring of the XiaF assay, we found evidence for the transient intermediate (denoted with an asterisk, Supplementary Fig. 2b,I; Fig. 3b,I), showing the expected exact mass (*m/z* [M-H]^−^ 364.1928 Da, calculated 364.1918 Da) (Supplementary Fig. 6). However, the proposed hydroxylated intermediate could not be detected *in vitro*.

### Phylogenetic relationship and genetic context of XiaF

To gain insight into the possible evolutionary origin of XiaF, we scrutinized related flavoenzymes, their catalytic function and biological role *in silico*. Through homology searches (BLAST), we compiled a diverse set of 28 XiaF homologues (Fig. 2b), most of which have been classified as group D flavin-dependent monooxygenases (FMOs). Group D FMOs are monooxygenases with an acetyl-CoA dehydrogenase (ACAD)-fold that receive reduced FAD/FMN from a NAD(P)H-dependent flavin reductase^35^. Various group D FMOs have been shown to catalyse aromatic hydroxylation and *N*-hydroxylation reactions^35^. Unexpectedly, apart from few secondary metabolite oxygenases, XiaF homologues identified in the homology search are involved in the catabolism of xenobiotics.

To investigate the phylogenetic position of XiaF, we used the neighbour-joining algorithm^36^ to construct a phylogenetic tree of all sequences from evolutionary distance data (Fig. 2b). XiaF forms a clade together with its orthologues from *S.* sp. SCSIO 02999 (XiaI) and *A. nigrescens* CSC17Ta-90 (XiaF′ #4; not yet characterized orthologues of XiaF were named XiaF′ and consecutively numbered from 1 to 6), as well as with five orthologues from other putative *xia* gene clusters from various actinomycetes. This subclade is part of a bigger clade including indigo-forming oxygenases pJEC, AcadA_pB7-2_ and IpoA discovered through soil metagenomics^27^,^37^,^38^. Although substrates other than indole are unknown for these three enzymes, the induction of their expression by artificial pollution (AcadA_pB7-2_)^27^ or the exposure to limonene (IpoA)^38^ implies a role in xenobiotic metabolism for at least two of them.

In the cladogram, we identified various additional groups of oxygenases that are specifically involved in xenobiotics degradation: dibenzothiophene monooxygenases from *Rhodococcus erythropolis* IGTS8 (DszC #1) and *Bacillus subtilis* (DszC #2)^39^,^40^; 4-nitrotoluene monooxygenases IcpA_clone M103_ and IcpA_clone M103_ discovered in a metagenome screening of activated sludge^41^; indole 3-acetic acid monooxygenases from *Acinetobacter baumannii* ATCC 19,606 (IacA #1) and *Pseudomonas putida* (IacA #2), and IdoA, for which the polycyclic aromatic hydrocarbon (PAH) fluoroanthrene was proposed to be the natural substrate^42^; 2-naphthoic acid monooxygenase NmoA^30^, 4-hydroxyphenylacetate 3-hydroxylase C2-HpaH^43^ and phenol monooxygenase PheA^44^.

**Figure 1:**
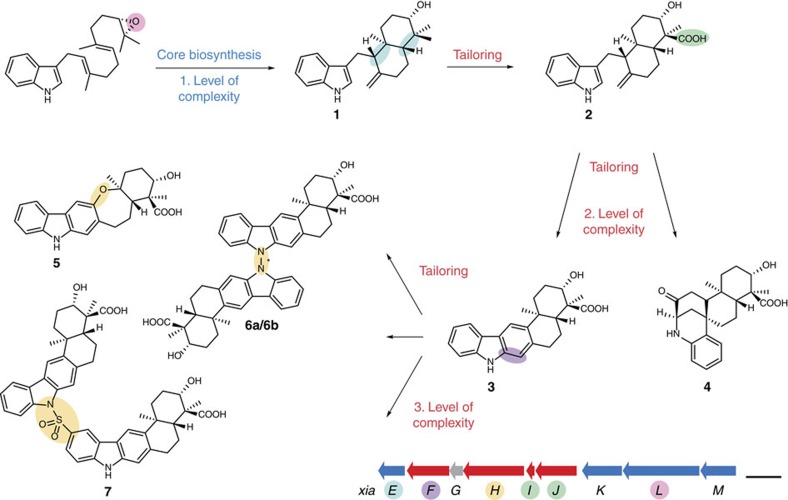
Model of bacterial IST biosynthesis. The biosynthetic scheme shows selected members of the xiamycin family of IST, their biogenetic relationship and their proposed biosynthetic pathway. IST biosynthesis comprises various levels of terpenoid diversification. An epoxidized linear IST-precursor has been proposed to be cyclized to yield preindosespene (**1**)^21^,^22^,^26^, which is subsequently oxygenated to the carboxylic acid indosespene (**2**)^22^,^46^. **2** has been proposed to be further cyclized into the spiro compound sespenine (**4**) or the carbazole ring system of xiamycin (**3**)^21^,^22^,^26^, of which the latter has been proposed to be further diversified to yield the cycloether oxiamycin (**5**)^18^,^23^, various *N,C*- and *N,N*-fused xiamycin dimers (such as **6a**/**6b**)^18^,^22^,^23^, and sulfonyl-bridged xiamycin dimers (such as **7**)^19^. Genes encoding enzymes that are involved in the core biosynthesis of the IST scaffold of preindosespene (**1**) are coloured in blue (*xiaEKLM*), genes for enzymes that tailor basic scaffolds to more complex ISTs are coloured in red (*xiaFGHIJ*) and genes with no proposed function are coloured in grey (*xiaG*). Atoms, bonds or functional groups that are added/are newly formed in biosynthetic reactions are highlighted in the same colour as the gene(s) encoding the gene product(s) that have been proposed to mediate these reactions^18^,^19^,^22^,^26^,^46^. The length of the scale bar represents a kilobase.

**Figure 2:**
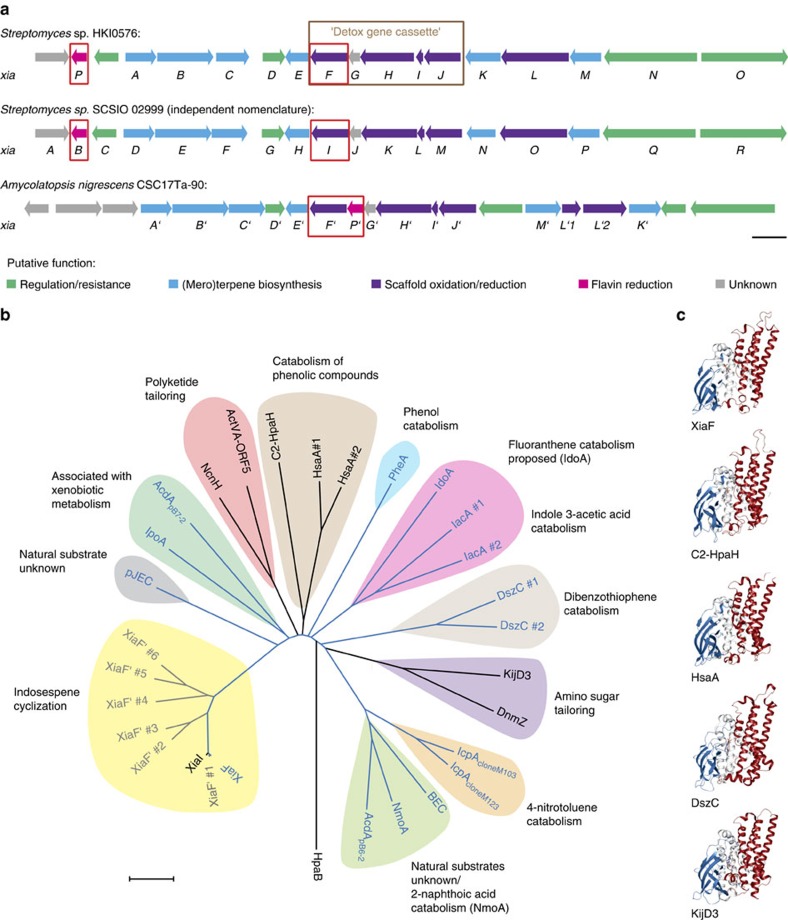
Comparison of *xia* gene loci, and phylogeny of XiaF and its homologues. (**a**) Comparison of the *xia* gene cluster of *Streptomyces* sp. (HKI0576) with the *xia* gene cluster of *Streptomyces* sp. SCSIO 02999 and a putative *xia* gene locus of *Amycolatopsis nigrescens* CSC17Ta-90. Genes for the two-component system XiaF/XiaP and orthologues are framed in red. The ‘detox gene cassette’ *xiaFGHIJ*, which encodes homologues of xenobiotic-metabolizing enzymes that are mostly involved in IST tailoring reactions is framed in brown. The length of the scale bar represents a kilobase. (**b**) Phylogenetic tree of XiaF and related flavoenzymes. Enzymes that have been reported to catalyse indigo formation are coloured in blue; putative XiaF orthologues of other putative *xia* gene clusters that yet have not been characterized are coloured in grey. The evolutionary history was inferred using the neighbour-joining method^36^. The oxygenase component HpaB of the 4-hydroxyphenylacetate 3-monooxygenase from *Thermus thermophilus* HB8 (ref. 90) was used as an outgroup. The length of the scale bar represents an evolutionary distance of 0.1 amino acid differences per site. (For further details, see the Supplementary Note 3 and Supplementary Figs 8–11.) (**c**) Structural comparison of XiaF (PDB ID: 5LVW), the 4-hydroxyphenylacetate 3-hydroxylase C2-HpaH (PDB ID: 2JBT)^48^, the secosteroid hydroxylase HsaA from *Mycobacterium tuberculosis* (PDB ID: 3AFE)^32^, the dibenzothiopene monooxygenase DszC from *Rhodococcus erythropolis* D-1 (PDB ID: 3X0Y)^52^ and the *N*-oxygenases KijD3 involved in D-kijanose biosynthesis (PDB ID: 4KCF)^53^. The structural alignment was calculated by the Dali server^51^ and shows the superposition of the different monomers. (For a more detailed structural comparison, see Supplementary Fig. 7.)

Surprisingly, also the genetic context of *xiaF* within the *xia* gene cluster of *S.* sp. (HKI0576) indicates its relationship to xenobiotics metabolism. On closer inspection, we found that *xiaF* forms a subcluster with genes (*xiaGHIJ*) that encode homologues of xenobiotic-metabolizing enzymes (Figs 1 and 2a). *XiaG*^22^ encodes a protein that is homologous to limonene‐1,2‐epoxide hydrolase^45^, whereas XiaH^18^,^19^,^22^ shares homology with flavoenzymes involved in the degradation of phenolic compounds^18^. The deduced gene product of *xiaJ* is a cytochrome P450 monooxygenase (CYP450)^22^,^46^, which is closely related to hydroxylases implicated in bacterial detoxification processes. Except for XiaG, all enzymes encoded in this subcluster are proposed to be involved in IST tailoring reactions^18^,^19^,^22^,^26^,^46^ (Fig. 1).

### XiaF catalyses a cryptic indole hydroxylation

**Figure 3:**
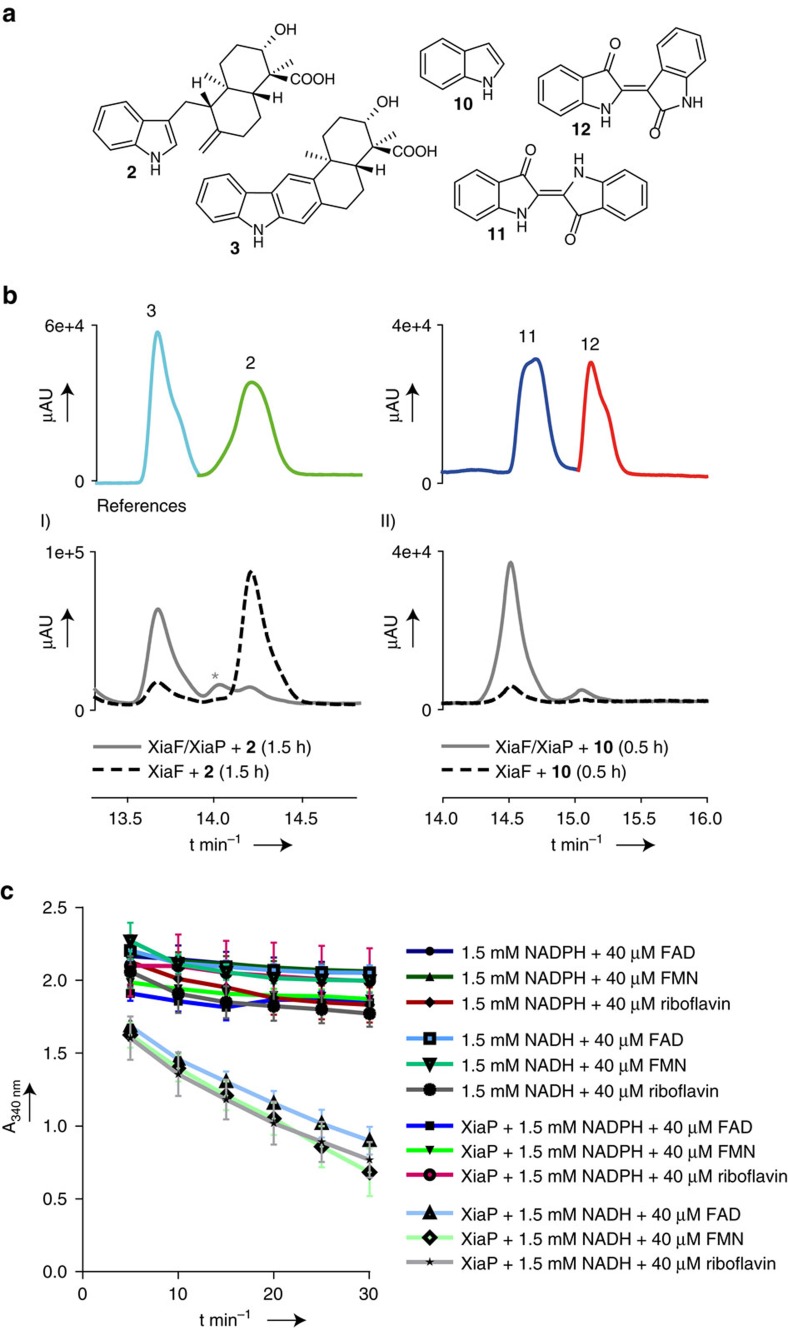
*In vitro* reconstitution and characterization of XiaF and XiaP. (**a**) Structures of substrates and products of XiaF enzyme assays. (**b**) HPLC-HRMS monitoring (PDA 300 nm) showing the results of (I) XiaF enzyme assays with indosespene (**2**) as the substrate in the presence and absence of XiaP (the asterisk denotes the proposed transient intermediate prexiamycin (**8**); for details, see Supplementary Fig. 6); (II) XiaF enzyme assays with indole (**10**) as the substrate in the presence and absence of XiaP. The profiles showing the reference compounds indosespene (**2**) and xiamycin (**3**) as well as indigo (**11**) and indirubin (**12**) are composed of different measurements of pure compounds (colour coded). (**c**) *In vitro* activity test of flavin reductase XiaP (0.1 μM). Conversion rates were followed by measuring the decrease in absorbance at 340 nm over time. Data represent mean values of three independent experiments, each conducted as duplicates. Error bars indicate s.d.

To test XiaF’s ability to detoxify typical xenobiotics, we added various aromatic compounds to the *in vitro* enzyme assay. We selected reported substrates of the XiaF homologues, 4-nitrotoluene, 4-hydroxyphenylacetate, 2-naphthoic acid, 3-hydroxybiphenyl, indole 3-acetic acid and indole. However, from this range of aromatic compounds, only indole (**10**) was transformed into an oxygenated product. LC-MS monitoring of the XiaF *in vitro* assay supplemented with indole gave diagnostic peaks for indigoid pigments indigo (**11**) and indirubin (**12**). Both compounds are known to result from oxidative dimerization of indoxyl (**13**), the immediate product of indole hydroxylation. Notably, these pigments were not formed in the negative control experiment using Fre and heat-denatured XiaF (Supplementary Fig. 2b,III). Likewise, no pigments could be detected in the chromatographic profile after 1.5 h incubation of XiaF alone; only when the assay was supplemented with the reductase partners Fre or XiaP, **11** as well as **12** were formed in considerable amounts (Fig. 3b,II and Supplementary Fig. 2b,IV). These results underpin that XiaF works in a two-component system together with a flavin reductase.

**Figure 4:**
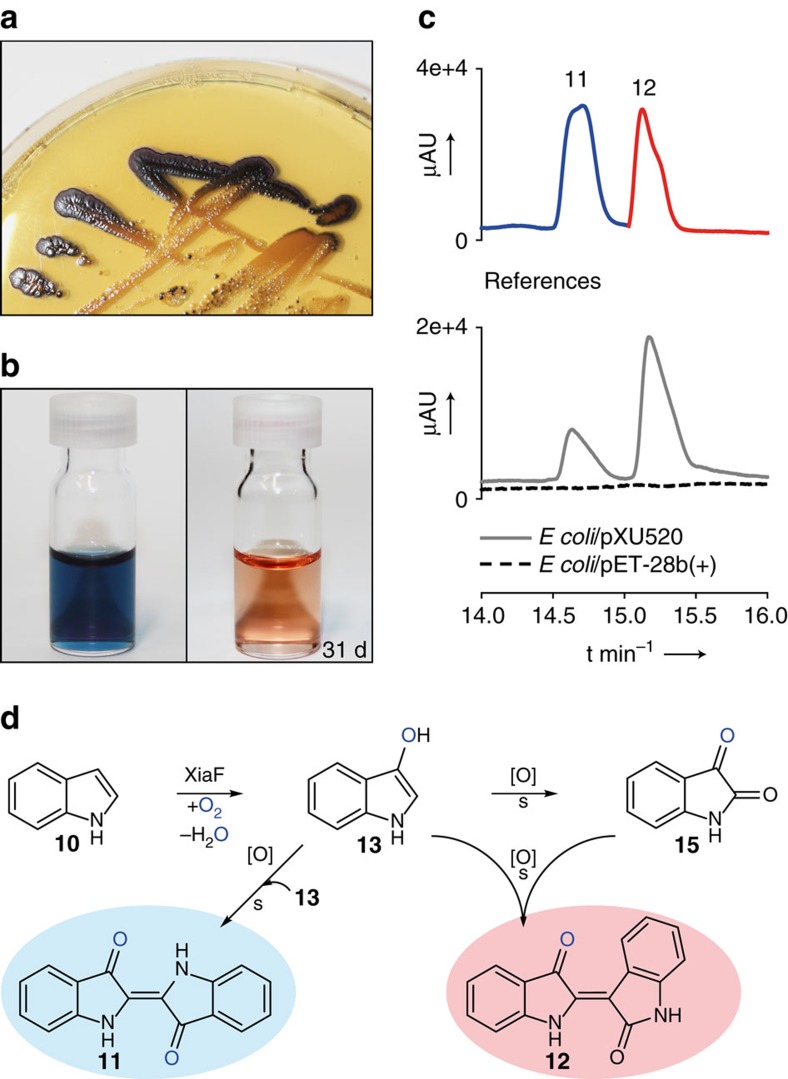
Indigo production catalysed by XiaF. (**a**) Blue pigment production of *xiaF*-expressing *E. coli* (*E. coli*/pXU520) plated on agar supplemented with tryptophan. (**b**) Dimethylformamide extract of *E. coli*/pXU520, which turns red after several days of incubation; reduction of indigo yields indigo white and reveals the red indirubin that is no longer overlaid by the dark blue indigo. (**c**) HPLC-HRMS monitoring (extracted ion chromatograms) of extracts from cultures of *E. coli*/pXU520. *E. coli* carrying the empty vector (*E. coli*/pET-28b(+)) was used as a negative control. The profiles showing the reference compounds indigo (**11**) and indirubin (**12**) are composed of different measurements of pure compounds (colour coded). (**d**) Hydroxylation of indole (**10**) catalysed by XiaF yields indoxyl (**13**), which is spontaneously oxidized to yield indigo (**11**). Alternatively, isatin (**15**) is formed from indoxyl as a side reaction, which can condense with indoxyl to yield indirubin (**12**), the structural isomer of **11**.

In addition to these *in vitro* experiments, we found that the same indigoid pigments are also formed *in vivo*. On extended incubation of *E. coli* plate cultures that harbour the *xiaF*-expression plasmid (*E. coli*/pXU520), we noted a slight colouration of the colonies. Blue pigment production was substantially enhanced when *E. coli*/pXU520 was plated on agar supplemented with tryptophan (Fig. 4a). Since the colouration appeared without coexpression of *fre* or *xiaP* in *E. coli*, XiaF obviously recruits native *E. coli* enzymes for flavin reduction. The blue pigmentation was completely absent in control strains harbouring the empty vector only. For a full characterization of the blue pigments, cells were grown in liquid tryptophan medium^47^. HPLC-HRMS analysis with authentic references unequivocally showed that the pigments are identical with indigo (**11**) and indirubin (**12**) (Fig. 4b,c).

### XiaF crystal structure and model of cryptic hydroxylation

**Figure 5:**
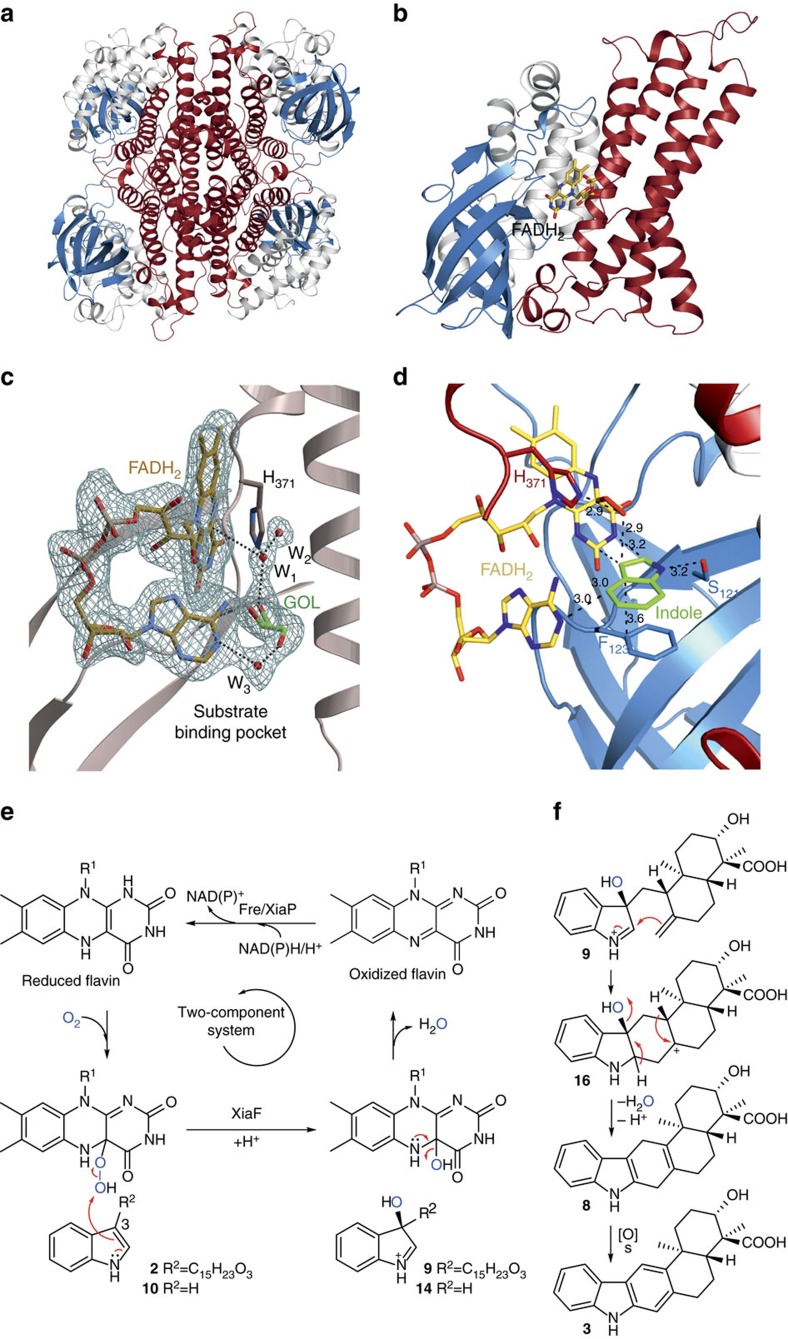
Structural analysis of XiaF and scheme of FADH_2_-mediated indole hydroxylation. (**a**) Ribbon structure of the homotetramer (PDB entry code: 5LVU). (**b**) Close-up cartoon representation of a monomer with bound FADH_2_ (PDB entry code: 5LVW) The N-terminal domain is coloured in white, the β-sheet domain in blue and the C-terminal domain in red. (**c**) Ribbon diagram of XiaF with the enlarged active site. The electron density represents a 2F_O_-F_C_ map with the displayed FADH_2_, glycerol (GOL) and three water molecules (W_1-3_) omitted for phasing (PDB ID: 5MR6). The amino acid is labelled by the one-letter code and numbered according to the XiaF sequence. Black dashed lines indicate hydrogen bonds. (**d**) Docking of indole (carbon atoms coloured in green), using AutoDock Vina^50^, with XiaF in complex with the modelled reaction intermediate FAD–OOH. Distances in Å. (**e**) Catalytic cycle of XiaF consuming one molecule of oxygen and of reduced flavin provided by cognate reductase, yielding a C-3-hydroxylated indole derivative. The oxidized cofactor is transferred back to the reductase where it is recycled in a NAD(P)H/H^+^-dependent reaction. (**f**) Nucleophilic attack of the *exo*-methylene group on 3-hydroxyiminium cation **9** results in the pentacyclic intermediate **16**. Deprotonation and loss of an equivalent of water yields prexiamycin (**8**), which aromatizes to yield **3**. Spontaneous reactions are indicated by ‘s’.

To further characterize the non-canonical terpenoid cyclase and the cryptic hydroxylation reaction, we carried out protein crystallization and modelling experiments. The structure of XiaF was solved at 2.4 Å resolution applying molecular replacement (*R*_free_=22.9%, search model: 4-hydroxyphenylacetate 3-hydroxylase, PDB ID: 2JBS^48^). XiaF (PDB entry codes: 5LVU, 5LVW and 5MR6) assembles as a tetrahedral oligomer, which is in agreement to the molecular mass of 176 kDa as determined by size-exclusion chromatography. The intermolecular interactions between the individual subunits are solely formed between the C-terminal domains, whereas the N-terminal as well as the central β-sheet domains are directed towards the corners of the tetrahedron (Fig. 5a). The N-domain spans from Met1-Lys118, the β-sheet from Leu119-Ser213 and the C domain from Gly214-Leu397 (Fig. 5b), which describes the typical architecture of a member of the ACAD superfamily (Supplementary Fig. 7).

Cocrystallizations of XiaF with flavin proved to be challenging; initially no FAD bound to crystals could be observed when the oxidized cofactor was used. A similar result has been reported for FMN and the XiaF homologue C2-HpaH (4-hydroxyphenylacetate 3-hydroxylase), which has been shown to bind reduced FMN considerably better than oxidized FMN^48^. Eventually, on incubation of XiaF with its cognate reductase XiaP under anaerobic conditions, we succeeded in binding reduced FADH_2_ to the active site (PDB entry code: 5LVW and 5MR6, Fig. 5c and Supplementary Fig. 12). This result corroborates the finding that XiaF needs a reductase that supplies the enzyme with reduced cofactor. The strong three-fold coordination of FADH_2_’s β-phosphate by Tyr144 (2.1 Å), Arg266 (2.0 Å) and Ala350 (3.0 Å) substantially contributes to the binding of the cofactor to the active site (Supplementary Fig. 14a). The hydrophobic part of the isoalloxazin ring is embedded within a hydrophobic pocket mainly comprised by Trp87 (3.1 Å), Tyr144 (4.2 Å) and Trp186 (3.8 Å). The ring N5 nitrogen is coordinated by the hydroxyl group of Thr146 (3.1 Å), whereas N3 and O4 are both stabilized by the carbonyl group of Ser121 (both 3.0 Å). Interestingly, the XiaF structure in complex with FADH_2_ and glycerol (PDB entry code: 5MR6) displays a unique bent conformation of the cofactor’s adenine moiety, thus blocking the substrate-binding channel. The phosphoribosyl linker is hereby exposed to the solvent (Fig. 5c and Supplementary Fig. 12). A similar conformation of FAD has been previously reported for an FAD synthase from yeast^49^. This bent conformation is missing the distinct interactions between the FADH_2_’s β-phosphate and the amino acids Tyr144, Arg266 and Ala350. Instead, three new intramolecular H-bonds between the ribose and adenine moieties of the cofactor are formed (3.0, 2.6 and 3.0 Å; Supplementary Fig. 14b). However, control assays with FMN, which lacks the adenine moiety, and the characterization of XiaF’s orthologue XiaI (ref. 21) have shown that FAD and FMN can both be used by the enzymes, a feature that is also shared by XiaF’s homologues C2-HpaH and HsaA from *Mycobacterium tuberculosis*^32^,^48^. Therefore, it is unclear whether this unique bent conformation has a biological function. As described previously for the XiaF homologue C2-HpaH^48^, the highly conserved His371 (3.7 Å from C4) sits above the FADH_2_ ligand, perfectly positioned for stabilizing, orienting and protonating the FAD-hydroperoxide intermediate. In course of the reaction, the distal electrophilic oxygen would be attacked by the double bond (C2–C3) of the indole moiety, thus leading to a hydroxylation of the C3 position. In case of the XiaF:FADH_2_:glycerol complex structure, the glycerol molecule exactly occupies the substrate position, as shown by a structural alignment with the XiaF homologue 4-hydroxyphenylacetate 3-hydroxylase (Supplementary Fig. 13, PDB ID: 2JBT). Furthermore, a water molecule is directly placed between the C4 of FADH_2_ (3.7 Å) and His371 (2.7 Å), indicating the position of the hydroperoxide (Fig. 5c and Supplementary Fig. 14b). Thus, we performed docking experiments using AutoDock Vina^50^ to place indole within the active site (Supplementary Fig. 15). This modelling highlights the role of Ser121 (3.2 Å) and Phe123 (3.6 Å) in positioning indole. Especially the π-stacking between the indole ring and Phe123 could be a key interaction catalyzing the specific hydroxylation, which ultimately leads to both indigo and xiamycin formation. Moreover, we used this structure to model the FAD–OOH reaction intermediate to highlight the hydroperoxide’s potential binding geometry relative to the indole ligand (Fig. 5d). This modelling approach suggests that the hydroperoxide is located between His371 (2.9 Å) and the C3 position of the indole ring (2.9 Å), thus explaining XiaF’s high specificity for hydroxylating indole.

A conservation mapping using the Dali server^51^ revealed that XiaF is structurally closely related to the 4-hydroxyphenylacetate 3-hydroxylase C2-HpaH (root mean square deviation of C_α_-carbon atoms (r.m.s.d.)=2.3 Å, PDB ID: 2JBT)^48^, the secosteroid hydroxylases HsaA from *Mycobacterium tuberculosis* (r.m.s.d.=1.8 Å, PDB ID: 3AFE)^32^, the dibenzothiophene monooxygenase DszC from *Rhodococcus erythropolis* D-1 (r.m.s.d.=2.6 Å, PDB ID: 3X0Y)^52^ and the *N*-oxygenase KijD3 involved in D-kijanose biosynthesis (r.m.s.d.=2.4 Å, PDB ID: 4KCF)^53^ (Fig. 2c; for a primary sequence alignment of XiaF and crystallized homologues, see Supplementary Fig. 10). The superposition of these homologous two-component monooxygenases reveals a strong structural conservation of the overall topology, whereas the individual substrate-binding pockets comprise a diverse set of amino acids (Supplementary Fig. 7). In case of the *N*-oxygenase KijD3, the overall substrate-binding channel is uniquely orientated compared to the above-mentioned homologous enzymes, highlighting the enzymes’ flexible active site architecture. It is noteworthy that the FADH_2_ coordinating residues are mainly located on the β-sheet domain, while the substrate-binding pocket forming amino acids predominantly derive from the C domain. This structural separation of the cofactor and the ligand-binding elements might theoretically allow a structural plasticity of the substrate-binding pocket without disturbing the fine-tuned cofactor binding. In case of XiaF, the substrate-binding pocket is comprised by residues N91, L98, S121, F123, I237, M240, H371 and M373 (Supplementary Fig. 7a).

## Discussion

In this study, we have gained insights into the structure, function and evolutionary relationship of a non-canonical terpenoid cyclase. Our findings illuminate the mechanism of the key step of this rare bacterial indoloterpenoid pathway, provide a biocatalyst for the production of indigoid pigments and shed more light on the evolution of metabolic diversity.

*In vitro* characterization, bioinformatics and structural investigations revealed that XiaF is a new member of the group D FMOs, monooxygenases that receive reduced FAD/FMN from a NAD(P)H-dependent flavin reductase and assemble in a tetrameric ACAD-fold composed of N- and C-terminal helical domains and a central β-sheet domain^35^. Genome mining and biochemical analyses revealed the designated flavin reductase partner of XiaF in a two-component system. The successful *in vitro* reconstitution of the two-component system has laid the foundation for studying bacterial indoloterpenoid biosynthesis in more detail. In the current model for xiamycin biosynthesis, it was proposed that a hydroxylated intermediate would set the basis for terpenoid cyclization^20^,^21^,^22^. While bioinspired synthetic studies support this model^25^,^54^, experimental evidence for an enzymatic hydroxylation as trigger for the cyclization reaction has been missing. Using indole as substrate surrogate, we unveiled the ‘cryptic hydroxylation’ at C-3 of the indole ring, which is clearly indicated by the formation of indigoid pigments. Hydroxylation of indole followed by deprotonation of intermediate **14** (Fig. 5e) results in the formation of indoxyl, which is spontaneously oxidized to yield indigo (Fig. 4d). Alternatively, in an oxygen-rich environment isatin (**15**) is formed from indoxyl as a side reaction. Condensation of indoxyl with isatin produces indirubin, which is a common by-product of indigo biosynthesis^55^. These findings have important implications for the XiaF-catalysed reaction mechanism. Accordingly, in the terpenoid pathway C-3 hydroxylation of the indole ring of indosespene by C4a-hydroperoxyflavin as the electrophilic oxygenating species yields a reactive intermediate (most likely the 3-hydroxyiminium cation **9**, although deprotonation to yield an hydroxyindolenine intermediate is also conceivable) that is prone to nucleophilic attack of the *exo*-methylene group, resulting in the pentacyclic intermediate **16** (Fig. 5f). Deprotonation and loss of an equivalent of water yields prexiamycin, which spontaneously aromatizes to yield **3**.

Beyond these important mechanistic implications, our findings are also relevant from a biotechnological point of view. Bacterial indigo production is of considerable interest as a sustainable and environmentally friendly substitute for the synthetic production of indigo that depends on coal and oil and generates toxic byproducts^56^,^57^,^58^,^59^. Also, the second indigoid pigment, indirubin, is a valuable natural product^58^. Indirubin is not only an antimicrobial agent^37^,^60^, but also has therapeutic value against diseases and pathological conditions like cancer, Alzheimer’s disease and inflammation^61^. In addition, indirubin is the main active ingredient of the plant mixture ‘Danggui Longhui Wan’, which has been used for millennia in traditional Chinese medicine to treat chronic diseases, likely because of its potent inhibition of cyclin-dependent kinases^62^,^63^. Because of these important bioactivities, recently there has been a renaissance of oxygenase screening activities towards indirubin production^37^,^47^,^60^,^64^,^65^. However, these enzymes generally seem to be rather promiscuous detoxification enzymes, not specialized indole oxygenases. In stark contrast, XiaF is to all appearance uniquely selective in hydroxylating the indole ring, since aromatic model xenobiotics have not been accepted as substrates. There seems to be the general trend that broad substrate acceptance comes at the price of low reaction turnover numbers^66^,^67^. Thus, a specialized indole oxygenase could be of particular interest for biotechnological applications, especially regarding large-scale indigoid pigment production. There is an unabated demand for indigo dye^57^, which is supplied by an annual world production of 80,000 tons of pigment^68^.

From an evolutionary point of view, the most intriguing discovery is that a terpenoid cyclase is functionally and structurally related to various enzymes from bacterial xenobiotic-degradation pathways. Xenobiotic-metabolizing enzymes have existed for more than 2.5 billion years^69^. Primordial cells have likely been exposed to many hostile chemical challenges and naturally occurring xenobiotics (for example, from volcano eruptions)^70^,^71^ and the requirement of protective mechanisms against cytotoxic and genotoxic chemicals are certainly older than secondary metabolism. Moreover, besides their role in detoxification, xenobiotic-metabolizing systems have evolved in organisms like bacteria and fungi to tap new chemical resources by degradation of foreign aromatic hydrocarbons^72^. Therefore, one may conceive a metabolic crosstalk of the myriad of ancient broad-specific detoxification/degradation enzymes that are constitutively emplaced to guard against xenobiotics and/or utilize alternative sources of energy and elements on endogenous biosynthetic pathways.

Indole hydroxylation and subsequent indigo formation is a side reaction of many xenobiotic-metabolizing enzymes^58^,^73^ that is often regarded as an indicator for general substrate tolerance towards aromatic hydrocarbons^27^,^41^,^74^. On the other hand, this reaction could also be the result of natural selection. In recent years, evidence is mounting that indole hydroxylation catalysed by these enzymes is of physiological relevance, since indole is toxic to microorganisms, and indigo formation by indole oxygenases represents a detoxification mechanism^75^,^76^,^77^. Moreover, the catalysis of indigo formation provides the missing link that connects XiaF with most of these catabolic oxygenases at a functional level.

On the basis of our structural data and docking experiments with indole, a potential binding mode of indole within the active site of XiaF could be revealed (Fig. 5d and Supplementary Fig. 13). A two-fold coordination of the substrate via an interaction of indole’s N1 and Ser121 as well as π-stacking between the aromatic substrate and Phe123 seems to be most important for molecular recognition of this chemical group. A modelling of the FAD–OOH reaction intermediate suggests a coordination sphere of indole, which places the hydroperoxide exactly above and in close distance to indole’s C3 position (Fig. 5d). This unique architecture may rationalize XiaF’s specificity for hydroxylating indole. From a structural point of view, the potential of XiaF to utilize highly diverse substrates like indole and indosespene is explained by the fact that only a small, conserved proportion of the ligand is bound to the active site, close to the highly reactive FAD–OOH group. The remaining part of indosespene is bound to an open substrate channel (Supplementary Fig. 7). In case of the XiaF homologue 4-hydroxyphenylacetate 3-hydroxylase, the active site is limited by Arg263 (ref. 48). This amino acid is replaced in XiaF by Ile237, which opens up the binding channel for accepting the elongated indosespene substrate. Thus, the flexible active site architecture of these enzymes presumably builds the basis for the enzymes’ potential utilization of various substrates.

We suggest that the promiscuous indole-metabolizing reaction, shared by many catabolic XiaF homologues might be the evolutionary origin of the cyclization reaction of indosespene (**2**). The most parsimonious explanation would be that XiaF has evolved from a broad-specific xenobiotic-metabolizing oxygenase that was able to accept the more complex indole compound **2**, besides its ability to metabolize indole. In contrast to the formation of indoxyl, which results in the formation of indigoid pigments, hydroxylation of **2** at position C-3 triggers cyclization. In the course of evolution, XiaF likely specialized on this particular reaction. This is indicated by experiments to test the substrate flexibility of XiaF, for which typical xenobiotic substrates of other XiaF homologues were not transformed.

Surprisingly, among the XiaF homologues are also the tentative polyketide-tailoring hydroxylases ActVA-ORF5 (ref. 78), and NcnH^79^ and the glycoside-modifying *N*-oxygenases KijD3 (ref. 53) and DnmZ^80^. Thus, it appears that various enzymes that catalyse secondary metabolite tailoring reactions could have evolved from detoxification pathways, yet this relationship has been overlooked.

Finally, our hypothesis that *xiaF* originates from xenobiotic metabolism is substantiated by the fact that it clusters with genes (*xiaGHIJ*) that encode homologues of xenobiotic-metabolizing enzymes. Therefore, it is tempting to speculate that this ‘detox gene cassette’ (Fig. 2a), which interrupts the core biosynthesis genes *xiaE* and *xiaKLM* originates from genes for broad-specific xenobiotic-metabolizing enzymes that were recruited for the diversification of the IST core scaffold preindosespene (**1**) (Fig. 1). The fact that xenobiotic-metabolizing enzymes are usually encoded on mobile genetic elements, such as transposons and plasmids, which are frequently shared by microorganisms via horizontal gene transfer within microbial communities^81^,^82^, together with the ability of these catabolic genomic islands to integrate into the chromosome after transfer^82^ makes such a scenario even more plausible. Overall, these considerations along with the structural and phylogenetic analyses, the biochemical characterization and biotransformation experiments indicate that the specialized terpenoid cyclase presumably has evolved from the functionally related detoxification enzymes.

## Methods

### Organisms, cultivation conditions and plasmids

The *E. coli* strains Top 10 (Life Technologies Corporation), BL21 (DE3) and BL21-CodonPlus(DE3)-RP (Agilent Technologies GmbH) were used for cloning and heterologous expression experiments. Electrocompetence of cells was achieved by repeated washing with diluted glycerol. Transformation was carried out in 2 mm cuvettes at 2.5 kV in a Gene Pulser Xcell electroporator (Bio-Rad) or according to the manual for commercially available chemical competent Top 10 cells (Life Technologies Corporation). Cells were grown in Luria broth (LB) medium, with appropriate antibiotics, whenever needed.

Plasmids used in this study were the pET-28a(+)-based expression vector (Merck KGaA) pET28-fre carrying a NAD(P)H-dependent flavin reductase gene (*fre*) combined with a 5′-sequence coding for a hexahistidine-tag, the pET-28b(+)-based expression vector pXU520 (for the production of recombinant XiaF with N-terminal histidine-tag), the pET-28c(+)-based expression vector pET28c-xiaP (for the production of recombinant XiaP with N-terminal histidine-tag) and the pET-26b(+)-based expression vector pET26b(+)-xiaF (for the production of recombinant XiaF with N-terminal histidine-tag used for crystallization).

### DNA preparation and analysis

PCR or digested products were separated on agarose gels and purified from the gel using innuPREP Gel Extraction Kit (Analytic Jena). Digestion and ligation reactions were carried out by standard methods. Isolation of plasmid DNA was accomplished with standard alkaline lysis principle.

### Construction of expression vectors for *xiaF*, *xiaP* and *fre*

For cloning the coding sequences of *xiaF* and *xiaP* downstream of the T7 promoter in pET28 vectors, the genes were PCR-amplified from cosmid 04b02 (from a genomic cosmid library of the endophyte) using primer sets XiaF_NdeI_fw (5′-GGAGTGGTGCATATGACAGA-3′, *Nde*I restriction site underlined) and XiaF_rv (5′-CGACATCGGGATGAGAAAG-3′) for *xiaF* and SK01 (5′-GCCATATGATCGACCGCGAGACCTTCG-3′) and SK02 (5′-CCGGATCCTCATCAACCGCCCGCCGGC-3′, *Nde*I and *Bam*HI restriction sites underlined) for *xiaP*. For crystallization of XiaF, the gene sequence was cloned into vector pET-26b(+). For that, *xiaF* was PCR-amplified using pXU520 as template and SK14 (5′-GATCATATGCATCATCACCACCACCATACAGACATCCGATCGGAG-3′) and SK15 (5′-CTAGGATCCTCATCAGAGGCTGGGCACGATGG-3′, *Nde*I and *Bam*HI restriction sites underlined) as primers. PCR was carried out with KAPAHiFi (PEQLAB) for *xiaF* and Phusion High-Fidelity DNA Polymerase (NEB) for *xiaP*. The amplicons were subcloned into the vector pCR-Blunt (Life Technologies) and the ligation mixture was introduced into electrocompetent *E. coli* Top 10 cells. Afterwards, plasmids were isolated from positive transformants and verified by sequencing. Excised DNA from correct plasmids was cloned by using the *Nde*I and *Bam*HI restriction sites of pET-28b(+) or pET-26b(+) (for recombinant *xiaF*) and pET-28c(+) (for recombinant *xiaP*) to generate pXU520 and pET26b(+)-xiaF (for the production of recombinant XiaF with N-terminal histidine-tag) and pET28c-xiaP (for the production of recombinant XiaP with N-terminal histidine tag). After introducing the ligation mixture into competent Top 10 cells, plasmids were isolated from positive transformants and verified by sequencing. Correct plasmids were introduced into *E. coli* BL21-CodonPlus(DE3)-RP for heterologous expression.

Amplification and cloning of *fre* from BL21 (DE3) were carried out as described by Zeng *et al*.^83^ leading to a pET-28a(+)-based expression vector (pet28a-fre), which was introduced into electrocompetent *E. coli* BL21-CodonPlus(DE3)-RP cells for overexpression.

### Production and purification of recombinant XiaF and XiaP

For heterologous protein production, *E. coli* BL21-CodonPlus(DE3)-RP carrying the plasmid pXU520 or pET26b(+)-xiaF for recombinant XiaF or pET28c-xiaP for recombinant XiaP were grown in LB medium supplemented with 34 μg ml^−1^ chloramphenicol and 50 μg ml^−1^ kanamycin at 30 °C and 180 r.p.m. to an OD_600_ of 0.4–0.5. After induction with 0.1 mM isopropyl-1-thio-*β*-D-galactoside (IPTG) for XiaF or 0.15 mM IPTG (for XiaP), cells were cultivated for 16 h at 15 °C and orbital shaking at 150 r.p.m. On collecting, the pellet from a 1.6-L-culture was resuspended in 50 ml cold XiaF lysis buffer (50 mM Tris-HCl pH 8.0, 500 mM NaCl, 10% glycerol, 0.2 mg ml^−1^ lysozyme, 110 μg ml^−1^ PMSF, 0.1% Triton X-100, 1 mM DTT) or XiaP lysis buffer (10 mM Tris-HCl pH 9.0, 100 mM NaCl, 10% glycerol, 0.3 mg ml^−1^ lysozyme, 0.5 mM PMSF, 0.1% Triton X-100, 1 mM DTT, 10 mM imidazole) and incubated for 30 min at 37 °C. Subsequently, the suspension was lysed by ultra-sonication with a Sonopuls HD 2,200 (BANDELIN). To separate cellular debris from soluble protein, the lysate was centrifuged.

For XiaF, soluble fraction was filtered (pore size 0.45 μm), diluted 1:1 with equilibration buffer (20 mM Tris-HCl pH 8.0, 200 mM NaCl, 10% glycerol, 1 mM DTT, 10 mM imidazole) and subjected to a FPLC machine (Äkta pure25, GE Healthcare Life Science) equipped with a Protino Ni-NTA column (Macherey*-*Nagel GmbH & Co. KG). His-tagged XiaF was eluted with 250 mM imidazole in the same buffer. Fractions containing XiaF were concentrated and buffer was exchanged against assay buffer (20 mM Tris-HCl pH 8.0, 10% glycerol) using ultrafiltration in Amicon Ultra-15 Centrifugal Filter (MWCO 3,000 Da) (Merck Millipore) at 22 °C before either treatment with thrombin to cleave the hexahistidine-tag or a size-exclusion chromatography was performed. For cleavage, 10 U thrombin were added per mg of XiaF and the reaction was incubated for 16 h at 4 °C in cleavage buffer (20 mM Tris-HCl pH 8.0, 150 mM NaCl, 25 mM CaCl_2_). The cleaved His-tag was separated by gravity-flow column purification with Protino Ni-NTA agarose resin (Macherey*-*Nagel GmbH & Co. KG). Thrombin was removed with benzamidine sepharose (GE Healthcare) in manual handling. Subsequently, tag-cleaved XiaF was concentrated in an Amicon Ultra-15 Centrifugal Filter (MWCO 3,000 Da) and dialysed with assay buffer at 22 °C. For size-exclusion chromatography (SEC) of His-tagged XiaF (44.5 kDa) and untagged XiaF (42.4 kDa), the protein solution was loaded onto a HiLoad 16/600 Superdex 200 prep grade column (GE Healthcare Life Science) by using FPLC. Elution was carried out with 1.5 column volumes (180 ml) of XiaF SEC buffer (20 mM Tris-HCl pH 8.0, 100 mM NaCl). Immediately on elution, XiaF fractions were supplemented with 10% glycerol to avoid precipitation, concentrated by ultrafiltration in Amicon Ultra-15 Centrifugal Filter (MWCO 3,000 Da) and dialysed with assay buffer. Calibration of SEC was performed according to the manual of Gel Filtration Calibration Kits (GE Healthcare), which implicated that XiaF natively occurs as a homotetrameric protein.

For XiaP, the supernatant was filtered (pore size 0.45 μm) and loaded onto a gravity-flow column filled with 3 ml of Protino Ni-NTA agarose resin (Macherey*-*Nagel GmbH & Co. KG) in a manual handling. Elution was obtained with elution buffer (10 mM Tris-HCl pH 9.0, 100 mM NaCl, 10% glycerol, 500 mM imidazole). Fractions containing XiaP were desalted with PD-10 columns (GE Healthcare) and resolved in XiaP storage buffer (100 mM NaH_2_PO_4_, 100 mM Na_2_HPO_4_, 10% glycerol, pH 7.0). For SEC of His-tagged XiaP, the protein solution was subjected onto a HiLoad 16/600 Superdex 200 prep grade column using a FPLC machine. Elution was carried out with 1.5 column volumes (180 ml) of XiaP SEC buffer (100 mM NaH_2_PO_4_, 100 mM Na_2_HPO_4_, 2% glycerol, pH 7.0). The protein was then concentrated in Amicon Ultra-15 Centrifugal Filter (MWCO 3,000 Da) and dialysed with XiaP storage buffer.

The purity of both proteins was checked on SDS–PAGE (Supplementary Fig. 1) and sequences were confirmed with MALDI-TOF-MS(MS) (UltrafleXtreme, Bruker) after in-gel tryptic digestion. For that, bands were excised and digested with Trypsin/Lys-C Mix (Promega) using the corresponding protocol. Short-term storage at 4 °C was possible for 3 days (XiaF) or 2 weeks (XiaP). For long-time storage, proteins were shock frozen in liquid nitrogen and stored at −80 °C.

### Production and purification of recombinant Fre

For overexpression of *fre*, a protocol based on Zeng *et al*.^83^ was used. The transformant (BL21-CodonPlus(DE3)-RP-pET28a-fre) was grown in LB medium supplemented with 25 μg ml^−1^ chloramphenicol and 35 μg ml^−1^ kanamycin at 30 °C and 200 r.p.m. orbital shaking to an OD_600_ of 0.4–0.6. Subsequently, cultures were induced with IPTG at a final concentration of 0.2 mM and incubated at 20 °C for 16 h. After collecting of cells, the pellet was resuspended in cold Fre lysis buffer (up to 50 ml for 1.6 l culture; 50 mM Tris-HCl pH 7.5, 150 mM NaCl, 1 mM EDTA, 2 mM DTT) and lysed by means of Sonopuls HD 2,200 (BANDELIN). Separated soluble fraction was subjected to 3 ml of Protino Ni-NTA agarose resin (Macherey*-*Nagel GmbH & Co. KG), and the mixture was shaken at 4 °C for 30 min before it was loaded into a gravity-flow column. Fre was eluted with 25–250 mM imidazole in Fre storage buffer (50 mM Tris-HCl pH 7.5, 300 mM NaCl, 1 mM EDTA, 2 mM DTT). Fractions containing Fre protein were concentrated using Amicon Ultra-15 Centrifugal Filter Units (MWCO 3,000 Da) (Merck KGaA) and dialysed with Fre storage buffer at 22 °C. The purity of His-tagged Fre (28.4 kDa) was checked on SDS–PAGE (Supplementary Fig. 1) and confirmed using in-gel tryptic digestion with MALDI-TOF-MS(MS). For long-term storage, protein was frozen at −80 °C in 50% glycerol.

### Activity assays of Fre and XiaP

The recombinant enzymes Fre (derived from *E. coli*) and XiaP (derived from *Streptomyces* sp. HKI0576) were tested for NAD(P)H:flavin oxidoreductase activity. Reactions were set up in XiaF assay buffer (20 μM Tris-HCl pH 8.0, 10% glycerol) containing 500 μM NAD(P)H (Sigma-Aldrich), 50 μM FAD/FMN/riboflavin (Sigma-Aldrich) and 1 μM flavin reductase at room temperature, unless stated otherwise. Reaction rates were monitored by following the decrease of NAD(P)H absorbance at 340 nm caused by the oxidation to NAD(P) on the Tecan Safire^2^ multi-detection microplate reader. Assays were carried out in a volume of 100 μl. Reactions without enzyme or flavin served as negative controls. Blanc measurements were performed with XiaF assay buffer und subtracted from each value.

### *In vitro* biotransformation assays with XiaF

To test the catalytic activity of XiaF, initial assays were performed with 25 μM XiaF, 2 mM NADH, 1 mM FAD and 100 μM indosespene in XiaF assay buffer at 28 °C for 24 h, which were supplemented with 10 μM Fre during the course of the study. Control assays contained heat-denatured XiaF (10 min at 99 °C). To study the influence of a reductase partner on the substrate conversion by XiaF assays were performed, which contained 500 μM NADH, 50 μM FAD, 100 μM indosespene and 10 μM XiaF and 10 μM Fre or 5 μM XiaF and 0.5 μM XiaP, in 100 μl or 50 μl total volumes. Reactions were carried out for at least 5 min at 28°C. Control assays contained no flavin reductase supplement. Extraction of the assay was performed three times with two volumes of ethyl acetate each time. The combined extracts were dried by evaporation of the solvent with nitrogen. The residues were dissolved in methanol and analysed with HPLC-HRMS (Exactive Plus Orbitrap Mass Spectrometer, Thermo Scientific) operating with a 150 × 2.1 mm 3 μ Betasil C18 column (Thermo Scientific) and a gradient from 5 to 98% solvent B in 15 min (solvent A, 0.3% (*v/v*) HCOOH in water; solvent B, 0.1% (*v/v*) HCOOH in acetonitrile) at a flow rate of 200 μl min^−1^ and UV/HRMS detection. As alternative substrates, indole (conditions identical to indosespene assays) and substrates from homologous enzymes (4-nitrotoluene, 4-hydroxyphenylacetate, 2-naphthoic acid, indole 3-acetic acid, 3-hydroxybiphenyl) were tested in XiaF biotransformation assays. In the latter case, reaction volumes were increased up to 300 μl. Maximum concentrations used were 50 μM XiaF, 10 μM XiaP and 2 mM substrates. For analysis, the reactions were stopped with one volume of acetonitrile (indole, 4-nitrotoluene) or methanol (indole 3-acetic acid), dried under reduced pressure, dissolved in acetonitrile or methanol and subjected to HPLC-HRMS. Reaction mixtures with 4-hydroxyphenylacetate, 2-naphthoic acid and 3-hydroxybiphenyl as substrates were extracted with ethyl acetate and analysed identical to indosespene assays. Control experiments were carried out without XiaF or with heat-denatured XiaF.

To test XiaF for flavin reductase activity reactions containing 15 μM XiaF, 500 μM NADH, 50 μM FAD and 100 μM indosespene and were performed at room temperature in XiaF assay buffer. Reaction rate was followed by the decrease in NADH absorbance at 340 nm on the Tecan Safire^2^ multi-detection microplate reader. Controls were carried out without XiaF or with Fre. Blanc measurements were performed with XiaF assay buffer and subtracted from each value.

### Indigoid pigment production *in vivo*

For pigment production on plate, an overnight culture of *E. coli* BL21-CodonPlus(DE3)-RP carrying the plasmid pXU520 (*E coli*/pXU520) was stroke out on LB-agar plates supplemented with 0.1 mM IPTG, 10 mM tryptophan and 50 μg ml^−1^ kanamycin. The plates were incubated at room temperature for several days until blue pigmentation occurred. *E. coli* BL21-CodonPlus(DE3)-RP carrying pET28(+) (*E coli*/pET-28b(+)) was used as a control. For pigment production in liquid culture, *E. coli*/pXU520 was grown in 100 ml tryptophan medium (2 g l^−1^ tryptophan, 5 g l^−1^ yeast extract, 10 g l^−1^ NaCl)^47^ supplemented with 50 μg ml^−1^ kanamycin at 30 °C and 200 r.p.m. After reaching an OD_600_ of 0.4, cells were induced with a final concentration of 0.5 mM IPTG and cultivated for 48 h. Centrifugation of the production culture resulted in a dark blue pellet. Extraction of this pellet with dimethylformamide yielded a dark blue extract, which turned reddish after several days of incubation. In addition, for pigment characterization, blue pellet was dried and extracted with acetonitrile. The extract was filtered and analysed by means of HPLC-HRMS (Exactive Plus Orbitrap Mass Spectrometer, Thermo Scientific) operating with a 150 × 2.1 mm 3 μ Betasil C18 column (Thermo Scientific) and a gradient from 5 to 98% solvent B in 15 min (solvent A, 0.3% (*v/v*) HCOOH in water; solvent B, 0.1% (*v/v*) HCOOH in acetonitrile) at a flow rate of 200 μl min^−1^ and UV/HRMS detection. Authentic references were used for comparison. *E. coli*/pET28b(+) was used as a control.

### Determination of kinetic parameters of XiaP

Flavin reductase (19,220 Da, sequence see below) activity was determined aerobically in XiaF assay buffer (20 mM Tris-HCl pH 8.0, 10% glycerol) at 28 °C in the dark. The reaction mixtures (100 μl or 120 μl) contained constant NAD(P)H and varying flavin concentrations (FAD, FMN, riboflavin), or *vice versa*, and 0.1 μM XiaP. His-tagged XiaP was purified in three independent experiments by affinity chromatography (IMAC) followed by a gel filtration technique (active appearance as homodimer). Concentrations of all XiaP solutions were determined and equalized using Pierce BCA Protein Assay Kit (Thermo Scientific). Assays were initiated by addition of enzyme. The activity was measured by monitoring the NAD(P)H absorbance at 340 nm in 96-well plates on a Varioskan LUX plate reader (Thermo Scientific). Initial decrease in NAD(P)H concentration over time was used to calculate the conversion rate (μM min^−1^ as *v*_0_). Apparent kinetic parameters were determined from double reciprocal plotting of initial velocities (*v*_o_^−1^) and substrate concentration ([S]_0_^−1^) and fitted by linear regression calculated with GraphPad Prism5. Three independent experiments with two technical replicates were used for calculation of kinetic parameters. For the determination of apparent kinetic values of NADH, 42 μM FMN or riboflavin were added to 4.1, 12.5, 25, 41.6, 58.3, 83.3, 208, 416, 833, 1,250, 1,500, 1,667, 2,083 and 2,500 μM of NADH (*V*_total_=120 μl) and absorbance was monitored for maximal 24 min. The apparent kinetic values of FAD, FMN or riboflavin were determined with varying concentrations of flavins (5, 10, 20, 40, 80, 100 and 150 μM) and 1.5 mM NADH. Absorbance measurement was started 5 min after enzyme addition for 25 min. For each absorbance value, buffer absorbance was subtracted and final absorbance was converted into NAD(P)H concentration by a calibration curve. Since autocatalytic NAD(P)H oxidation in control assays (without XiaP) was minute in comparison to the XiaP enzyme assays (Supplementary Fig. 4a,d) its influence was neglected for the determination of the kinetic parameters of XiaP. Moreover, the impact of the different kinds of flavins on the absorbance was considered to be equal and was neglected for the determination of the kinetic parameters of XiaP as well. Flavins that were putatively bound to XiaP after purifications (noticeable by a very slight yellow colour of the solution) were neglected in the determination since control measurements of XiaP with added NAD(P)H did not lead to a decrease in absorbance as well as none of the heat-denatured enzyme solutions revealed a detectable absorbance at 450 nm.

Amino-acid sequence of His-tagged XiaP used for determination of kinetic parameters: MGSSHHHHHHSSGLVPRGSHMIDRETFVELMSGVCAPVTVVTTATADGRPHGSTVSSFTSLSLDPPLASFALDRGSGLLPHLHQGARVGVNILAAHQHALAASFARRCPGTGSKFDGITWTTRAGLPHLPDSAGWTAGRVERHVP GGDHVLLVISVEEAHSTPAAPLV YLRRSFGTYSGLPAGG.

### Phylogenetic analysis

The evolutionary history was inferred using the neighbour-joining method^36^ and tested with the bootstrap test (1,000 replicates)^84^. The tree (Fig. 2b) is drawn to scale, with branch lengths in the same units as those of the evolutionary distances used to infer the phylogenetic tree. The evolutionary distances were computed using the p-distance method^85^ and are in the units of the number of amino-acid differences per site. Evolutionary analyses were conducted in MEGA6 (ref. 86). The analysis involved the following enzymes (NCBI/PDB accession number in brackets): XiaF from *Streptomyces* sp. HKI0576 (CCH63732)^22^, XiaI from *Streptomyces* sp. SCSIO 02999 (AFK78075)^21^, XiaF′ #1 from *Streptomyces olivaceus* (WP_031042714)^26^, XiaF′ #2 from *Streptomyces* sp. NRRL S-15 (WP_031097971)^26^, XiaF′ #3 from *Streptomyces fradiae* (WP_043467134)^26^, XiaF′ #4 from *Amycolatopsis nigrescens* CSC17Ta-90 (WP_020673280)^26^, XiaF′ #5 from *Streptomyces* sp. AA0539 (Streptomyces sp. AA0539)^26^, XiaF′ #6 from *Streptomyces* sp. NRRL F-2890 (WP_030727840)^26^, pJEC from uncultured bacterium JEC54 (AAY54299)^37^, IpoA from *Rhodococcus* sp. T104 (AAS88875)^38^, AcdA_pB7-2_ (BAP28583)^27^, AcdA_pB6-2_ (BAP28542)^27^, NcnH from *Streptomyces arenae* (AAG44124)^79^, ActVA-ORF5 from *Streptomyces coelicolor* A3(2) (WP_011030043)^33^, C2-HpaH from *Acinetobacter baumannii* (Q6Q272)^43^, HsaA #1 from *Mycobacterium tuberculosis* (WP_003900101)^32^, HsaA #2 from *Rhodococcus jostii* RHA1 (Q0S811)^34^, PheA from *Geobacillus stearothermophilus* (AAA85688)^44^, IdoA from *Pseudomonas alcaligenes* PA-10 (AF474963)^42^, IacA #1 from *Acinetobacter baumannii* ATCC 19,606 (EEX04702)^87^, IacA #2 from *Pseudomonas putida* (ABY62757)^88^, DszC #1 from *Rhodococcus erythropolis* IGTS8 (AAA99484)^39^, DszC #2 from *Bacillus subtilis* (BAC20182)^40^, KijD3 from *Actinomadura kijaniata* (4KCF_A)^53^, DnmZ from *Streptomyces peucetius* (4ZXV_A)^80^, IcpA_clone M103_ (BAH29956)^41^, IcpA_clone M123_ (BAH29955)^41^, BEC from *Cupriavidus necator* (AAK38074)^89^, NmoA from *Burkholderia* sp. JT1500 (CAD97420)^30^ and HpaB from *Thermus thermophilus* HB8 (WP_011228328) as an outgroup^90^. The result of the phylogenetic tree was confirmed with the minimal evolution method^91^, which was used as an alternative method. For more detailed cladograms ,see Supplementary Figs 8 and 9.

### Crystallization and structure determination

Crystals of XiaF were grown at 20 °C within 2 days to their final size applying the hanging drop (1 μl × 1 μl) vapour diffusion method. For XiaF:FADH_2_ crystals, XiaF (127 μM), XiaP (79 μM), NADH (500 μM) and FAD (150 μM) were anaerobically mixed inside a glove box and incubated for 25 min before setting up the crystallization trials. The XiaF:FADH_2_ crystals appeared in 1.6 M (NH_4_)_2_SO_4_, 100 mM HEPES/NaOH, pH 7.5 and 100 mM NaCl. Subsequently, crystals were transferred in 4 μl drops containing the crystallization buffer plus 30% of glycerol. After 2 min, crystals were vitrified and stored in liquid nitrogen. For XiaF:FADH_2_:glycerol crystals, XiaF (193 μM), XiaP (80 μM), NADH (2.75 mM), FAD (480 μM) and Na-dithionite (20 mM) were mixed and incubated for 60 min at 10 °C. Next, 1.1 mM of indosespene was added and incubated for 15 min. This mixture was used for setting up the crystallization trials. The XiaF:FADH_2_:glycerol crystals appeared in 1.6 M (NH_4_)_2_SO_4_, 100 mM HEPES/NaOH, pH 7.5, 100 mM NaCl and 5 mM Na-dithionite. Subsequently, crystals were transferred in 4 μl drops containing the crystallization buffer plus 30% of glycerol and 5 mM of Na-dithionite. Diffraction data sets were recorded using synchrotron radiation of *λ*=1.0 Å at the beamline X06SA, Swiss Light Source (SLS), Paul Scherrer Institut, Villigen, Switzerland. Collected reflections were processed using the programme package XDS. The statistics are summarized in  Supplementary Table 3. Determination of the crystal structure was performed by molecular replacement using the programme PHASER. Coordinates of 4-hydroxyphenylacetate 3-hydroxylase (C2-HpaH) (PDB ID: 2JBR)^48^ were applied as a suitable Patterson search model for the XiaF (apo) structure elucidation. The refined coordinates of XiaF in turn were employed for the XiaF structure in complex with FADH_2_ as well as the complex with FADH_2_ and glycerol. Notably, successful processing of the latter data set was only possible in the space group P1. Model building has been carried out with the graphic programme MAIN^92^ and finalized applying REFMAC5 (ref. 93) by conventional crystallographic rigid body, positional and anisotropic temperature factor refinements. Water molecules were placed automatically in the 2F_O_-F_C_-electron density maps (contoured at 1σ) using the software package ARP/wARP^94^. The achieved crystal structures possess crystallographic values of at least *R*_free_=22.9%, r.m.s.d. bond lengths of 0.012 Å, and r.m.s.d. bond angle of 1.6° (Supplementary Table 3). The crystal structures were deposited at the RCSB Protein Data Bank under the accession codes 5LVU for XiaF (apo), 5LVW for XiaF (FADH_2_) and 5MR6 for XiaF (FADH_2_+glycerol).

### Docking of the indole ligand into the active site of XiaF

For docking of the indole ligand, the XiaF structure in complex with FADH_2_ and glycerol was chosen (PDB ID: 5MR6). The position of the glycerol ligand served as search centre for the docking experiment. The docking grid was restricted to the active site, which is limited by FADH_2_’s adenine moiety. The indole ligand was built and energetically minimized using the Avogadro software^95^. The docking simulation was performed using AutoDock Vina^50^ as a plugin from the structure analysis software suite Chimera^96^. To prepare XiaF for the docking experiment, the plugin Dock Prep from the Chimera software suite was used. Hereby, solvent molecules were deleted and Gasteiger charges and hydrogens were added. For the latter ones, hydrogen bonds were considered as well, non-polar hydrogens were merged. Next, AutoDock Vina was run. Therefore, hydrogens were added to the indole ligand as well. The search was centred around the glycerol ligand coordinates (*x*=49.1286, *y*=−47.6138, *z*=−0.4335), the docking grid had a size of 10.0586 Å × 9.5815 Å × 7.8537 Å. For the receptor (XiaF), all of the following options were selected to be true: Merge charges and remove non-polar hydrogens, merge charges and remove lone pairs, ignore waters, ignore chains of non-standard residues, ignore all non-standard residues. For the ligand (indole), the following settings were selected to be true: Merge charges and remove non-polar hydrogens, merge charges and remove lone pairs. The number of binding modes was set to 10, the exhaustiveness of search was set to 8 and the maximum energy difference (kcal mol^−1^) was set to 3. In summary, 10 different conformations were computed, ranking from −5.3 (best hit) to −4.8 kcal mol^−1^ (worst hit). The best hit was used for subsequent XiaF analysis (Fig. 5d and Supplementary Fig. 15). The choice to use this specific indole conformation was not solely based on its docking score, but also considering its orientation relative to the H_2_O molecule W_2_ (Fig. 5c), which presumably complies with the orientation of the hydroperoxide group during catalysis. The second best score, for example, flips the ligand vertically, increasing the distance between the C3 position of indole and W_2_. The XiaF:FADH_2_:glycerol structure docked with indole was further analysed by modelling the reaction intermediate FAD–OOH using the graphic programme MAIN^92^ (Fig. 5d).

### Data availability

The coordinates and structure factors of XiaF have been deposited in the Protein Data Bank (http://www.rcsb.org/pdb) with accession numbers 5LVU, 5LVW and 5MR6. Any additional data not shown in this published article or in its Supplementary Information are available from the corresponding authors on reasonable request.

## Additional information

**How to cite this article:** Kugel, S. *et al*. Cryptic indole hydroxylation by a non-canonical terpenoid cyclase parallels bacterial xenobiotic detoxification. *Nat. Commun.* **8,** 15804 doi: 10.1038/ncomms15804 (2017).

**Publisher’s note:** Springer Nature remains neutral with regard to jurisdictional claims in published maps and institutional affiliations.

## Supplementary Material

Supplementary Information Supplementary Figures, Supplementary Tables, Supplementary Notes and Supplementary References
