# Supplementary material for: Cryptic indole hydroxylation by a non-canonical terpenoid cyclase parallels bacterial xenobiotic detoxification
Source: Nat Commun. 2017 Jun 15;8:15804. doi: 10.1038/ncomms15804 (PMC5481743; doi:10.1038/ncomms15804)
Supplement: Supplementary Information — Supplementary Figures, Supplementary Tables, Supplementary Notes and Supplementary References [file ncomms15804-s1.pdf]

Supplementary Notes

Supplementary Note 1. *In vitro* Reconstitution of XiaF using Flavin Reductase Fre from *E. coli*

Many XiaF homologs identified in this study have been reported to be part of a two-component systems consisting of a flavin-dependent oxygenase and a NAD(P)H:flavin oxidoreductase (flavin reductase)[1-7]. To test whether the poor *in vitro* performance of XiaF could be overcome by addition of a flavin reductase we initially tested Fre, the flavin reductase from *E. coli*, which has successfully substituted flavin reductase partners in other two-component systems[8-10]. In the presence of Fre, XiaF reproducibly converted indosespene (**2**) to xiamycin (**3**). In the negative control experiment using denatured XiaF, no product was detectable (Supplementary Figure 2A, I). With this robust enzyme assay at hand, we set out to investigate the influence of the reductase partner on the conversion of indosespene by XiaF in more detail. We conducted assays with reduced concentration of enzymes and cofactors in the presence and absence of Fre and found that Fre substantially enhances the activity of XiaF. Whereas in the assay lacking Fre most of the substrate (**2**) remained unconverted, after 3 h incubation **2** was fully converted into **3** when adding the reductase (Supplementary Figure 2B, II). Similar observations were made when indole (**10**) was used as a substrate in XiaF *in vitro* assays (Supplementary Figure 2B, III and IV). We next evaluated the ability of XiaF to catalyze flavin reduction through oxidation of pyridine nucleotides. Compared to the positive control with Fre, in which NADH/H^+ got rapidly oxidized, NADH/H^+ depletion with XiaF alone was comparable to the buffer (negative) control. Thus, XiaF alone is not sufficient to catalyze flavin reduction (Supplementary Figure 3). Therefore, we suggest that autocatalytic flavin reduction[11] in the presence of reduced pyridine nucleotides yielded traces of reduced flavin and led to the basal activity of XiaF in initial assays lacking Fre. This autocatalytic flavin reduction might also explain the basal activity of the XiaF ortholog XiaI in assays with extended incubation times and high cofactor concentrations[12,13].

Supplementary Note 2. Kinetic Analysis of XiaP

Determination of flavin reductase activity revealed, that XiaP is specific for NADH, whereas NADPH seems to be no suitable cofactor. In contrast, NADH was used by XiaP with all tested flavins (FAD, FMN, riboflavin) (Figure 3C). Determination of the apparent K_M value (K_M^{app}) for flavins showed that XiaP seems to have no marked substrate preference: With a K_M^{app} value of 3 µM for FMN, 5 µM for riboflavin and 9 µM for FAD all K_M^{app} values are in a comparable range (Supplementary Table 1). Determination of the apparent kinetic parameters of NADH with either FMN or riboflavin as constant second substrate led to K_M^{app} values of either 223 or 248 µM, respectively (Supplementary Table 2). Control experiments without XiaP revealed almost no autocatalytic NADH oxidation (Supplementary

Figure 4A). Therefore, its influence was neglected in the calculation of the kinetic parameters. As an example for the determination of initial velocities (v_0) Supplementary Figure 4B depicts the decrease in NADH concentration in reactions with varying riboflavin concentrations. Results were similar to calculations according to Michaelis-Menten equation (representative plots in Supplementary Figure 4C and 4D).

Supplementary Note 3. Detailed Phylogenetic Analysis of XiaF

Homology searches (BLAST) resulted in a diverse set of (28) XiaF homologs (Figure 2B), most of which have been classified as group D flavin-dependent monooxygenases (FMOs). Various group D FMOs have been shown to catalyze aromatic hydroxylation and *N*-hydroxylation reactions[14]. Unexpectedly, the majority of XiaF homologs is involved in the catabolism of xenobiotics, and not in secondary metabolite biosynthetic pathways, and shares the ability to catalyze indole hydroxylation to yield indigo with the indosespene cyclase XiaF[1,2,15-25]. Notably, none of them has been reported to use indole as its 'natural' substrate. Instead, indole is reported to be accepted as an alternative substrate or to be co-metabolized together with the natural substrates[1,2,16,18-21,23-25]. However, the concept to distinguish between natural and alternative substrate for promiscuous xenobiotic-metabolizing enzymes is questionable, especially in the light of recent studies, which suggest that the conversion of indole to indigo is a detoxification mechanism of microorganisms to deal with otherwise toxic indole[26-28].

Among the relatives of XiaF there are only four enzymes that have been implicated in tailoring of secondary metabolites. However, none of them has been reported to catalyze the production of indigo as an alternative reaction, or to be evolutionarily related to xenobiotic-metabolizing enzymes.

To investigate the phylogenetic position of XiaF we constructed a phylogenetic tree of all 30 sequences using the Neighbor-Joining method (Figure 2B). XiaF forms a clade together with its orthologs from *S.* sp. SCSIO 02999 (XiaI) and *A. nigrescens* CSC17Ta-90 (XiaF' #4), as well as with six orthologs from other putative *xia* gene clusters from various actinomycetes. This subclade is part of a bigger clade including the indigo-forming oxygenases pJEC, $AcadA_{pB7\text{-}2}$ and IpoA. The former two were identified in the course of soil metagenome screening projects. Whereas pJEC was discovered because of its ability to form indigoid pigments[15], $AcdA_{pB7\text{-}2}$ was identified from a soil sample that was artificially polluted with aromatic compounds (biphenyl, phenanthrene, carbazole, and 3-chlorobenzoate)[24]. Such metagenome screenings, which make use of the fact that several types of oxygenases are able to produce indigo when heterologously produced in suitable hosts, are frequently used to find potential biocatalysts for the oxygenation of aromatic compounds[23,24,29]. IpoA has been proposed to be involved in limonene degradation, since *ipoA* expression together with indigo formation is specifically induced when administering limonene[25]. Likewise, the induction of

AcadA$_{pB7-2}$ and IpoA production by xenobiotics strongly suggests their involvement in xenobiotic metabolism, although their natural substrates are still unknown.

We identified various additional clades of oxygenases that are mainly involved in xenobiotic catabolism. These are a) the dibenzothiophene monooxygenases from *Rhodococcus erythropolis* IGTS8 (DszC #1) and *Bacillus subtilis* (DszC #2)[2,20]; b) the 4-nitrotoluene monooxygenases IcpA$_{clone\ M103}$ and IcpA$_{clone\ M103}$ that have been discovered in a metagenome screening from samples of activated sludge used to treat coke plant wastewater[23]; c) the indole 3-acetic acid monooxygenases from *Acinetobacter baumannii* ATCC 19606 (IacA #1) and *Pseudomonas putida* (IacA #2), and IdoA for which the polycyclic aromatic hydrocarbon (PAH) fluoroanthrene was proposed to be the natural substrat[18]; and d) the 2-naphthoic acid monooxygenase NmoA[1], which clades together with BEC[17], for which no substrate is known except of indole, and AcdA$_{pB6-2}$, which was discovered in the same screening as AcdA$_{pB7-2}$[24].

Another clade is constituted by secosteroid hydroxylases from *Mycobacterium tuberculosis* (HsaA #1) and *Rhodococcus* sp. Rha1 (HsaA #2), which catalyze an important step in the degradation sequence of cholesterol[3,4], and the 4-hydroxyphenylacetate 3-hydroxylase C2-HpaH[5]. Notably, these oxygenases are closely related to the bifunctional hydroxylase ActVA-ORF5, which is involved in the biosynthesis of actinorhodin[30] and NcnH, which is presumably involved in naphthocyclinone biosynthesis[31]. All above-mentioned enzymes seem to be related to the phenol monooxygenase PheA[16]. All together, it appears that various secondary metabolite tailoring enzymes share an evolutionary origin with enzymes involved in xenobiotic-metabolizing/detoxification pathways, yet this relationship has been overlooked for them. In addition to these polyketide hydroxylases, XiaF homologs also comprise *N*-oxygenases KijD3[32] and DnmZ[7] involved in the biosynthetic pathways to kijanimicin[32], and baumycin[7], respectively.

All homologs of XiaF have been functionally characterized to a different extent except of the putative XiaF orthologs of other putative *xia* gene clusters that are uncharacterized yet.

PheA from *Geobacillus stearothermophilus* has been shown *in vivo* and *in vitro* (crude enzyme extract) to catalyze the conversion of phenol to catechol in the degradation pathway of phenol. Furthermore PheA has been reported to catalyze the conversion of indole to indigo and also to be active on cresols[16]. PheA is supported by the flavin reductase PheA2[33].

DszC from *Rhodococcus erythropolis* IGTS8 (DszC #1) has been characterized *in vitro* to catalyze the conversion of dibenzothiophene to dibenzothiophene sulfone in the degradation pathway of dibenzothiophene (DBT), which results in the formation of 2-hydroxybiphenyl[34]. DszC #1 is supported by the NADH:FMN oxidoreductase DszD[35].

DszC from *Bacillus subtilis* (DszC #2) has been characterized *in vivo* to catalyze the conversion of dibenzothiophene to dibenzothiophene sulfone[36]. Moreover, *in vivo* experiments to test the

substrate specificity indicate that DszC #2 is also active on 2,8-dimethylDBT, 4,6-dimethylDBT, and 3,4-benzoDBT[36]. Furthermore, DszC #1 and #2 have been shown *in vivo* (in *E. coli*) to catalyze the production of indigo[2,20].

NmoA from *Burkholderia* sp. JT1500 has been shown *in vitro* to catalyze the hydroxylation of 2-naphthoate to 1-hydroxy-2-naphthoate in a proposed 13-step 2-naphthoate degradation pathway. Moreover, NmoA has been shown to catalyze the conversion of indole to indigo *in vitro*[1]. NmoA works together with the NADH:flavin oxidoreductase NmoB[1].

IacA from *Acinetobacter baumannii* ATCC 19606 (IacA #1) has been shown *in vitro* to catalyze the conversion of indole to indigo. Furthermore, a mutagenesis study indicated that IacA #1 is involved in the catabolism of the plant hormone indole 3-acetic acid (IAA), which is used as an energy source[21]. Crude extracts from *E. coli* overexpressing *iacA* have been shown to oxidize IAA[21] and 2-hydroxy-IAA has been suggested to be the product of the IacA reaction[37].

IacA from *Pseudomonas putida* (IacA #2) has been shown *in vivo* (in *E. coli*) to catalyze the production of indigo. Furthermore, IacA has been suggested to be involve in the catabolism of IAA.[19]

IdoA from *Pseudomonas alcaligenes* PA-10 has been shown *in vivo* (in *E. coli*) to catalyze the production of indigo. Furthermore, the polycyclic aromatic hydrocarbon (PAH) fluoranthene has been suggested as a substrate of IdoA after a *idoA* gene deletion mutant had lost the ability to degrade this compound[18].

IpoA from *Rhodococcus* sp. T104 has been shown *in vivo* (in *E. coli*) to catalyze the production of indigo. Moreover IpoA has been suggested to take part in the degradation of limonene since *ipoA* expression together with indigo formation is specifically induced by feeding of limonene[25].

$IcpA_{cloneM103}$ and $IcpA_{cloneM123}$, which have been discovered in a metagenomic approach aiming to find biocatalytic enzymes from samples of activated sludge used to treat coke plant wastewater, have been shown *in vivo* (in *E. coli*) to catalyze the production of indigo. Furthermore $IcpA_{cloneM103}$ has been shown to catalyze the hydroxylation of 4-nitrotoluene to 4-nitrobenzyl alcohol *in vivo* (in *E. coli*)[23]. Both IcpA have been suggested to work together with the flavin reductase IcpB[23].

C2-HpaH from *Acinetobacter baumannii* has been characterized *in vitro*[5]. Moreover its crystal structure has been solved[38]. This enzyme catalyzes the hydroxylation of 4-hydroxyphenylacetate at C-3 resulting in the formation of 3,4-dihydroxyphenylacetate in the degradation pathway of phenolic compounds and works together with the flavin reductase C1-HpaH[5]. *In vitro* C2-HpaH has also been shown to accept 3-(4-hydroxyphenyl)propionate, 4-hydroxybenzoate and 4-nitrophenol as alternative substrates[39].

The secosteroid hydroxylases HsaA from *Mycobacterium tuberculosis* (HsaA #1) been characterized *in vitro*. Moreover its crystal structure has been solved. HsaA #1 catalyzes the hydroxylation of 3-hydroxy-9,10-seconandrost-1,3,5(10)-triene-9,17-dione (3-HSA) to the dihydroxy derivative 3,9-

dihydroxy-9,10-seconandrost-1,3,5(10)-triene-17-one (3,9-DHSA) in the degradation pathway of cholesterol and works together with the flavin reductase HsaB[3]. *In vitro* HsaA #1 also accepts 3,17-dihydroxy-9,10-seconandrost-1,3,5(10)-triene-9-one (3,17-DHSA), 3,9-dihydroxy-9,10-seconandrost-1,3,5(10)-triene-17-one (3,9-DHSA) and than 3-hydroxybiphenyl as alternative substrates and converts these compounds to the corresponding catechols[3].

HsaA from *Rhodococcus* sp. Rha1 (HsaA #2)[4] has been characterized by mutational analysis. A gene deletion of *hsaA* in *Rhodococcus* sp. Rha1 resulted in the accumulation of 3-HAS. Addition of HsaA from *Mycobacterium tuberculosis* culture supernatant of the rhodococcal Δ*hsaA* mutant resulted in the formation of 3,9-DHSA, supporting the proposal of HsaA as the hydroxylase of 3-HAS in *Rhodococcus* sp. Rha1 as well as in *Mycobacterium tuberculosis*[4].

All confirmed and proposed substrates from the above mentioned XiaF homologs (phenol, dibenzothiophene, 2-naphthoate, IAA, fluoranthene, limonene, 4-nitrotoluene, 4-hydroxyphenylacetate and 3-HSA) are xenobiotics or intermediates of the degradation of xenobiotics in the metabolizing organisms, which are derived from man-made or natural pollution as well as the secondary metabolism of other organisms.

Artificial pollution of soil with the four aromatic compounds biphenyl, phenanthrene, carbazole, and 3-chlorobenzoate, led to the discovery of $AcdA_{pB6-2}$ and $AcdA_{pB7-2}$ in a metagenomal screening. Both enzymes, together with different unrelated oxygenases that seem to be involved in the degradation of recalcitrant aromatic compound have been identified due to their ability to catalyze the production of indigo *in vivo* (in *E. coli*)[24]. $AcdA_{pB6-2}$ and $AcdA_{pB7-2}$ haven't been further characterized.

A metagenomic screening with forest soil also led to the discovery of pJEC due to its ability to catalyze the production of indigo *in vivo* (in *E. coli*). This enzyme has not been characterized in detail, either[15].

BEC has been shown *in vivo* to catalyze the production of indigo after blue-pigmented *E. coli* transformants occurred when a genomic library of *Ralstonia eutropha* HF39 was constructed[17]. BEC has not been biochemically characterized.

The sugar *N*-oxygenases KijD3 has been characterized *in vitro*[40]. Moreover its crystal structure has been solved[32,40]. KijD3 has been shown to produce a hydroxylamino species in the biosynthesis of the nitro-containing sugar D-kijanose[40]. KijD3 has also been proposed to catalyze further oxidation of the hydroxylamino species to D-kijanose, although this reaction has never been observed *in vitro*[32,40]. The putative NAD(P)H-dependent flavin reductase KijD6 has been proposed to assist KijD3[41]. For the *in vitro* characterization of KijD3 the flavin reductase Fre from *E. coli* was used[40].

The sugar *N*-oxygenases DnmZ has been characterized *in vitro*[7]. Moreover its crystal structure has been solved[42]. DnmZ has been shown *in vitro* to trigger oxidative carbon–carbon bond cleavage of the thymidine diphosphate (TDP)-functionalized amino sugar TDP-L-*epi*-vancosamine to yield an

aldehyde−oxime species in the biosynthesis of baumycins *via* a nitroso intermediate[7]. For the *in vitro* characterization of DnmZ a flavin reductase from *Vibrio* has been used[7].

ActVA-ORF5 has been characterized *in vivo* and *in vitro*[30,43]. ActVA-ORF5 is involved in the biosynthesis of actinorhodin and has been proposed to convert the dihydroxynaphthalene partial structure of 6-deoxy-dihydrokalafungin (DDHK) to its tetrahydroxynaphthalene form by the sequential hydroxylation of DDHK at C-6 and C-8[30]. The ability of ActVA-ORF5 to catalyze C-6 oxygenation has been shown *in vitro* by using emodinanthrone as a model substrate[30,43]. Moreover, the C-8 oxygenation activity of ActVA-ORF5 has been shown *in vivo* by complementation of an engineered *Streptomyces coelicolor* strain that expressed the minimal gene set for the production of DDHK[30]. ActVA-ORF5 works together with the flavin:NADH oxidoreductase ActVB[30,43].

NcnH, a putative hydroxylase encoded in the naphthocyclinone biosynthetic gene cluster has been shown *in vivo* to hydroxylate the dihyroxymethylanthraquinone aloesaponarin II to hydroxyaloesaponarin after heterologous expression in an engineered *Streptomyces coelicolor* strain that expressed various polyketide core producing genes[31].

For a summary of the reactions catalyzed by homologs of XiaF see Supplementary Figure 11.

Supplementary Figures

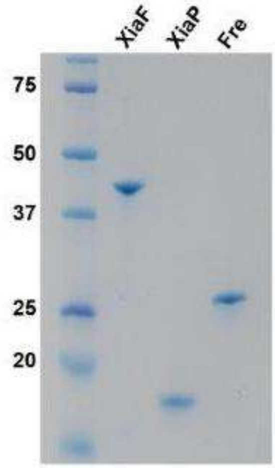

Supplementary Figure 1. SDS-Gel of Recombinant Proteins
Recombinant XiaF (42.4 kDa), XiaP (19.2 kDa) and Fre (28.4 kDa) on an Any kD™ TGX SDS-Gel (Bio-Rad), molecular weight standard Precision Plus Protein™ Dual Color Standards (Bio-Rad), molecular weight in kDa.

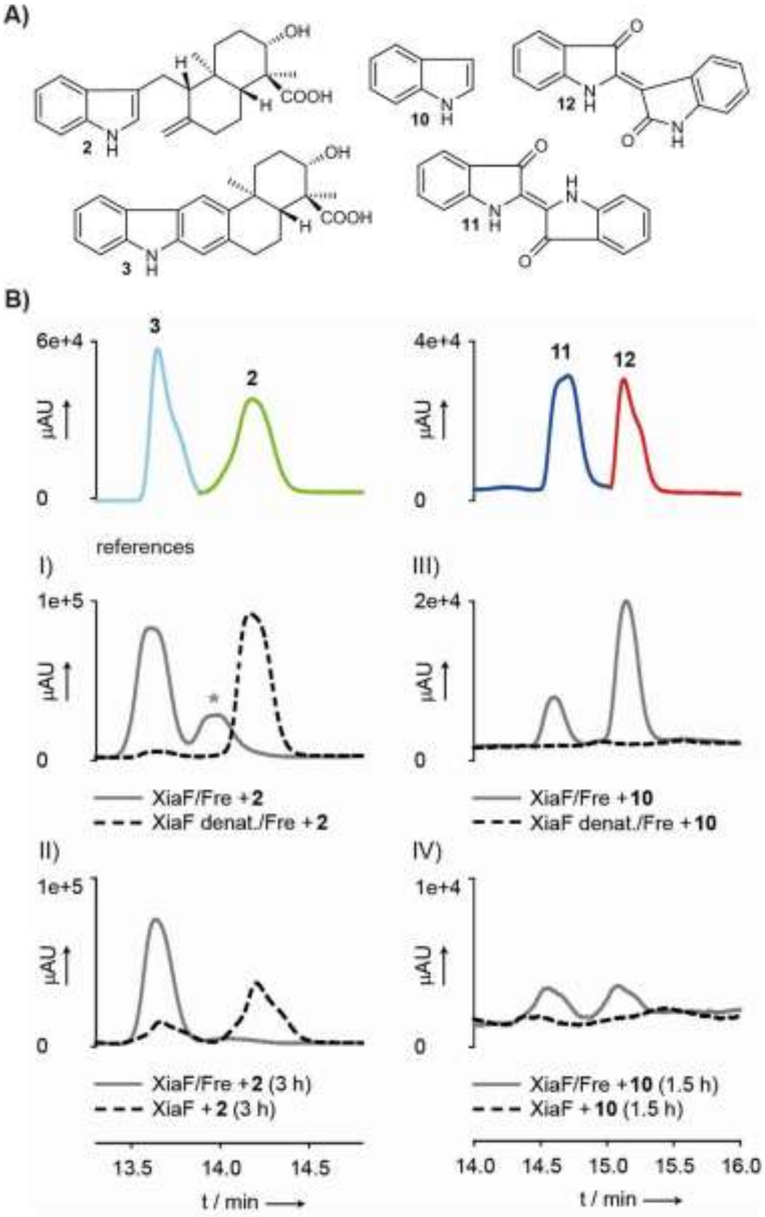

Supplementary Figure 2. ***In vitro* Reconstitution of XiaF and Fre**

A) Structures of substrates and products of XiaF enzyme assays. **B)** HPLC-HRMS monitoring (PDA 300 nm) showing the results of I) XiaF enzyme assays in the presence of Fre with indosespene (**2**) as the substrate and denatured XiaF as a control (the asterisk denotes the proposed transient intermediate prexiamycin (**8**); for details see Supplementary Figure 6); II) XiaF enzyme assays with **2** as the substrate in the presence and absence of Fre; III) XiaF enzyme assays in the presence of Fre with indole (**10**) as the substrate and denatured XiaF as a control; IV) XiaF enzyme assays with **10** as the substrate in the presence and absence of Fre. The profiles showing the reference compounds indosespene (**2**) and xiamycin (**3**) as well as indigo (**11**) and indirubin (**12**) are composed of different measurements of pure compounds (color-coded).

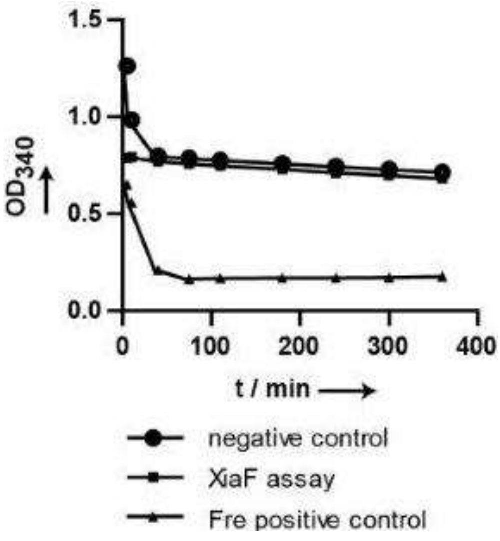

Supplementary Figure 3. Test of XiaF for Flavin Reductase Activity
Reactions composed of 15 µM XiaF, 500 µM NADH, 50 µM FAD, 100 µM indosespene (**2**). Compositions lacking XiaF served as negative control, reactions with 10 µM Fre as positive control. Conversion rate was monitored by measuring the absorbance at 340 nm over time. Data shown are representative single values.

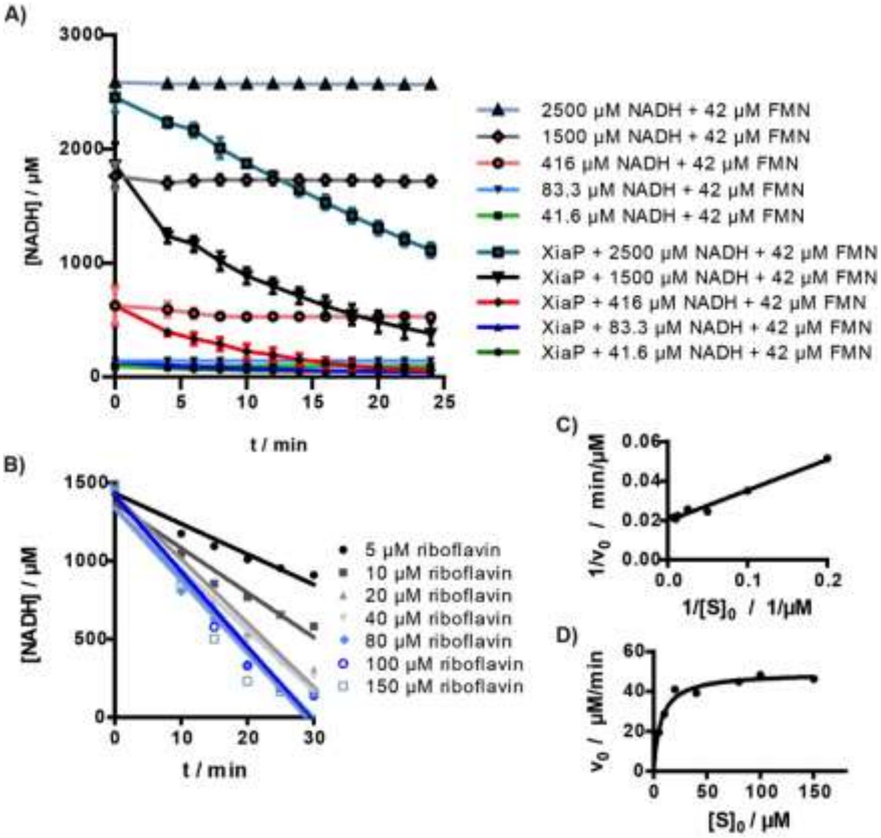

Supplementary Figure 4. Determination of Kinetic Parameters of XiaP

A) NADH oxidation activity tests without or with XiaP (0.1 µM). Varying NADH and constant FMN concentrations were used to study the influence of autocatalytic NADH oxidation. Conversion rates were followed by measuring the decrease in absorbance at 340 nm over time. By means of calibration the absorbances were transferred into values of NADH concentration. Data represent mean values of three independent experiments, each conducted as duplicates. Error bars indicate standard deviations. B-D) A replicate example of the reaction of 0.1 µM XiaP with 1.5 mM NADH and varying riboflavin concentrations. B: Conversion rates were monitored and absorbances were converted into concentration values as stated in A). Initial velocity rates (v_0 in µM min^{-1}) were calculated from fitting a linear regression curve into each reaction course and used for the determination of kinetic parameters from a double reciprocal plot (in C)) or Michaelis-Menten curve (in D)).

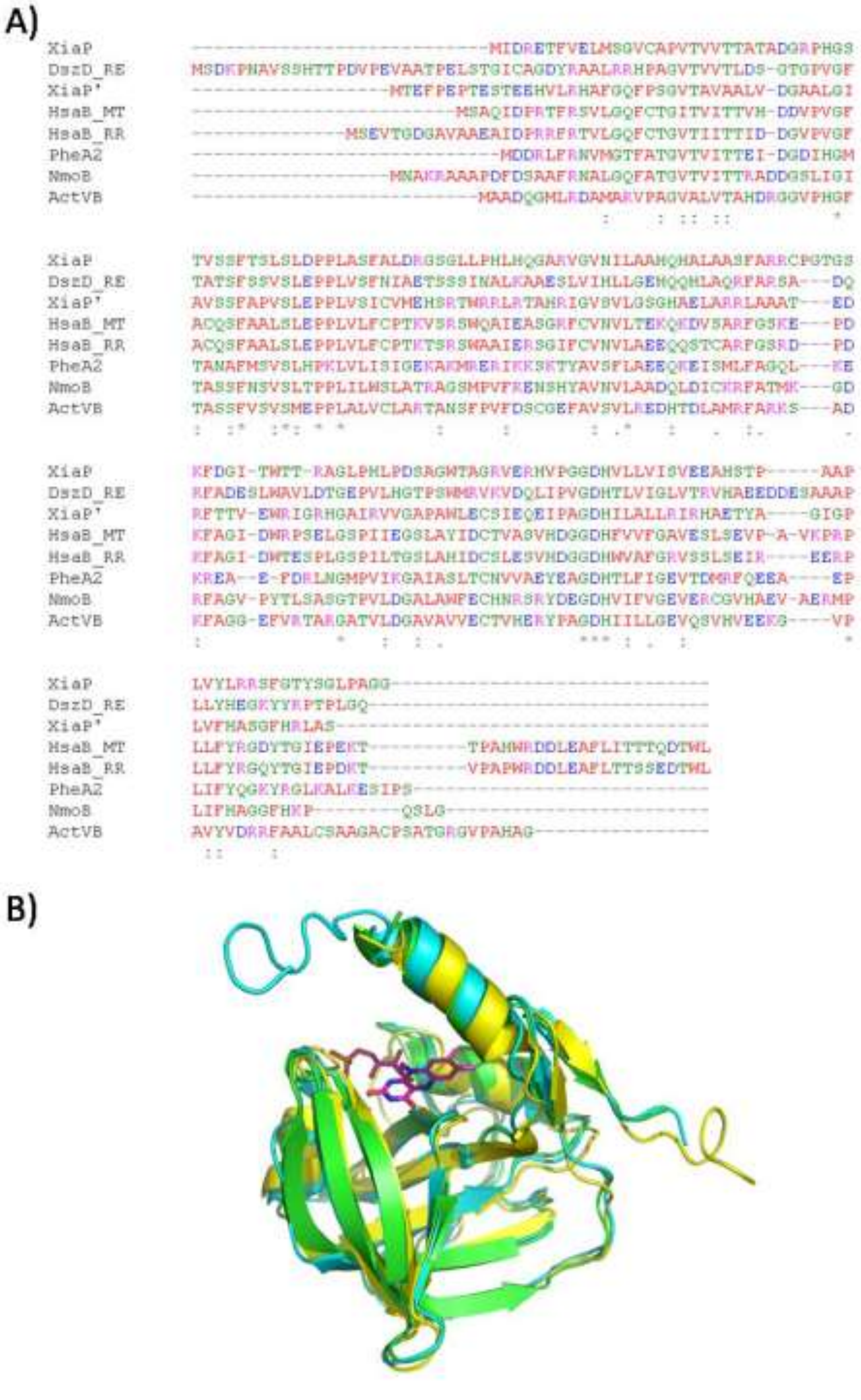

Supplementary Figure 5. Primary Sequence and Structural Alignments of Related Reductases
Homology of XiaP and XiaP' from *Amycolatopsis nigrescens* CSC17Ta-90 with known flavin reductase partner of XiaF homologs (Figure 2B). A) Alignment of XiaP and XiaP' with PheA2[33], NmoB[1], DszD from *Rhodococcus erythropolis* IGTS8[44] (Dsz_RE), HsaB from *Mycobacterium tuberculosis*[3] (HsaB_MT) and *Rhodococcus* sp. Rha1[4] (HsaB_RR) and ActVB[6]. The alignment was made with Clustal Omega[45]. B) Structural alignment of models of XiaP (yellow) and XiaP' (blue) that were predicted by I-TASSER[46] and the crystal structure of DszD (green) from *Mycobacterium goodii* (PDB ID: 3PFT). The ribbon diagrams have been prepared with PyMol[47].

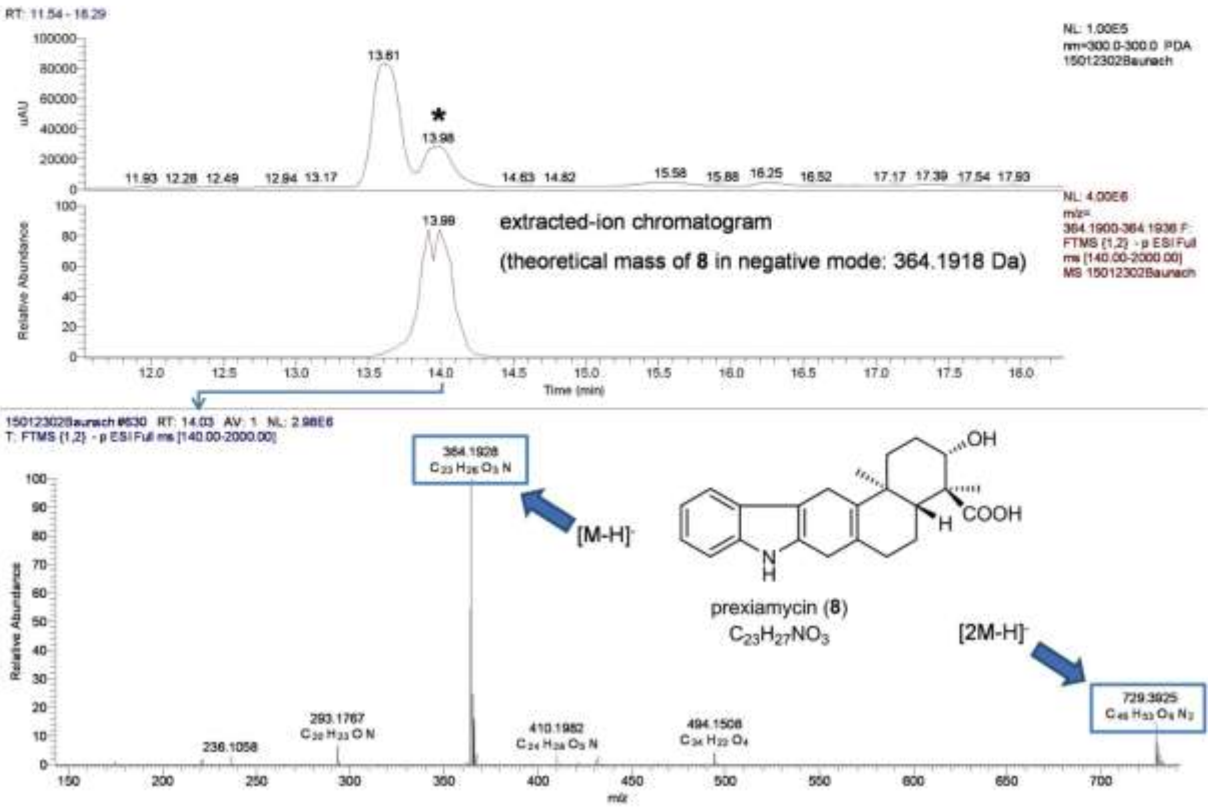

Supplementary Figure 6. Spectrometric Evidence for the Transient Intermediate Prexiamycin (8)

HPLC-HRMS monitoring (PDA 300 nm) of a XiaF enzyme assay in the presence of Fre with indosespene (**2**) as the substrate (Supplementary Figure 2B, I) shows another product that accompanies the biosynthesis of xiamycin (**3**) (around 14 min, denoted with an asterisk). HRMS analysis (extracted-ion chromatogram, negative mode) of the product revealed an exact mass of *m/z* [M-H]$^-$ 364.1928 Da, which matches the calculated mass of **8** (*m/z* [M-H]$^-$ 364.1918 Da).

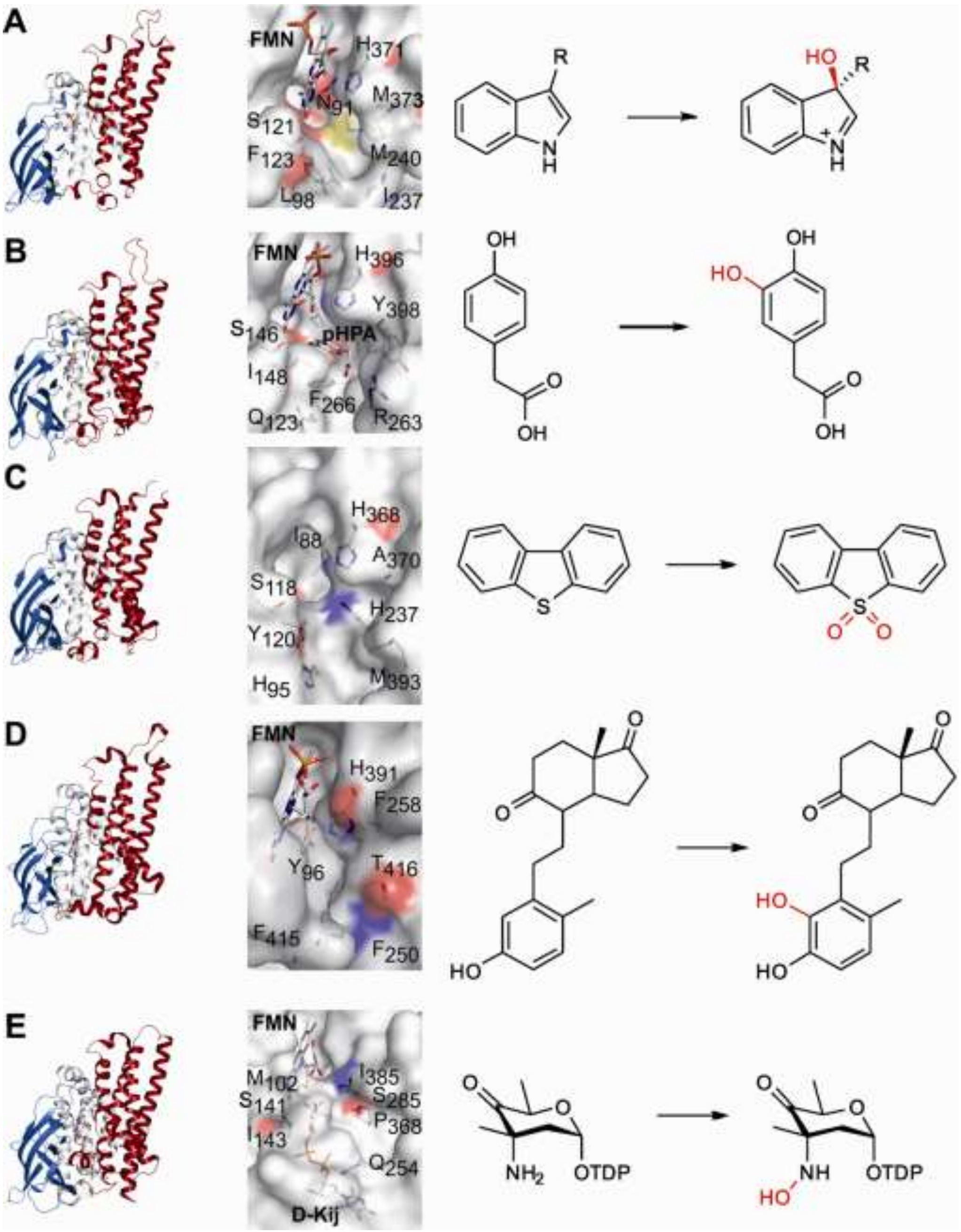

Supplementary Figure 7. Structural and Functional Comparison of XiaF and Homologous Enzymes

Shown are XiaF (A, PDB ID: 5LVW), the 4-hydroxyphenylacetate 3-hydroxylase C2-HpaH (B, PDB ID: 2JBT), the secosteroid hydroxylase HsaA from *Mycobacterium tuberculosis* (C, PDB ID: 3AFE), the dibenzothiophene monooxygenase DszC from *Rhodococcus erythropolis* D-1 (D, PDB ID: 3X0Y), and the *N*-oxygenases KijD3 involved in D-kijanose biosynthesis (E, PDB ID: 4KCF). The structural alignment was calculated by the DALI server [48]. The first column shows the superposition of the different monomers, the middle column displays a close-up view of the substrate-binding pocket, and the last column summarizes the reaction catalyzed by the individual flavin-dependent monooxygenases. The substrate-binding pockets of the different monooxygenases comprise the following residues: A) N91, L98, S121, F123, I237, M240, H371 and M373, B) Q123, S146, I148, R263, H396, Y398, C) I88, H95, S118, Y120, H237, H368, A370, M393, D) Y96, F250, F258, H391, F415, T416, E) M102, S141, I143, Q254, S285, P368, I385.

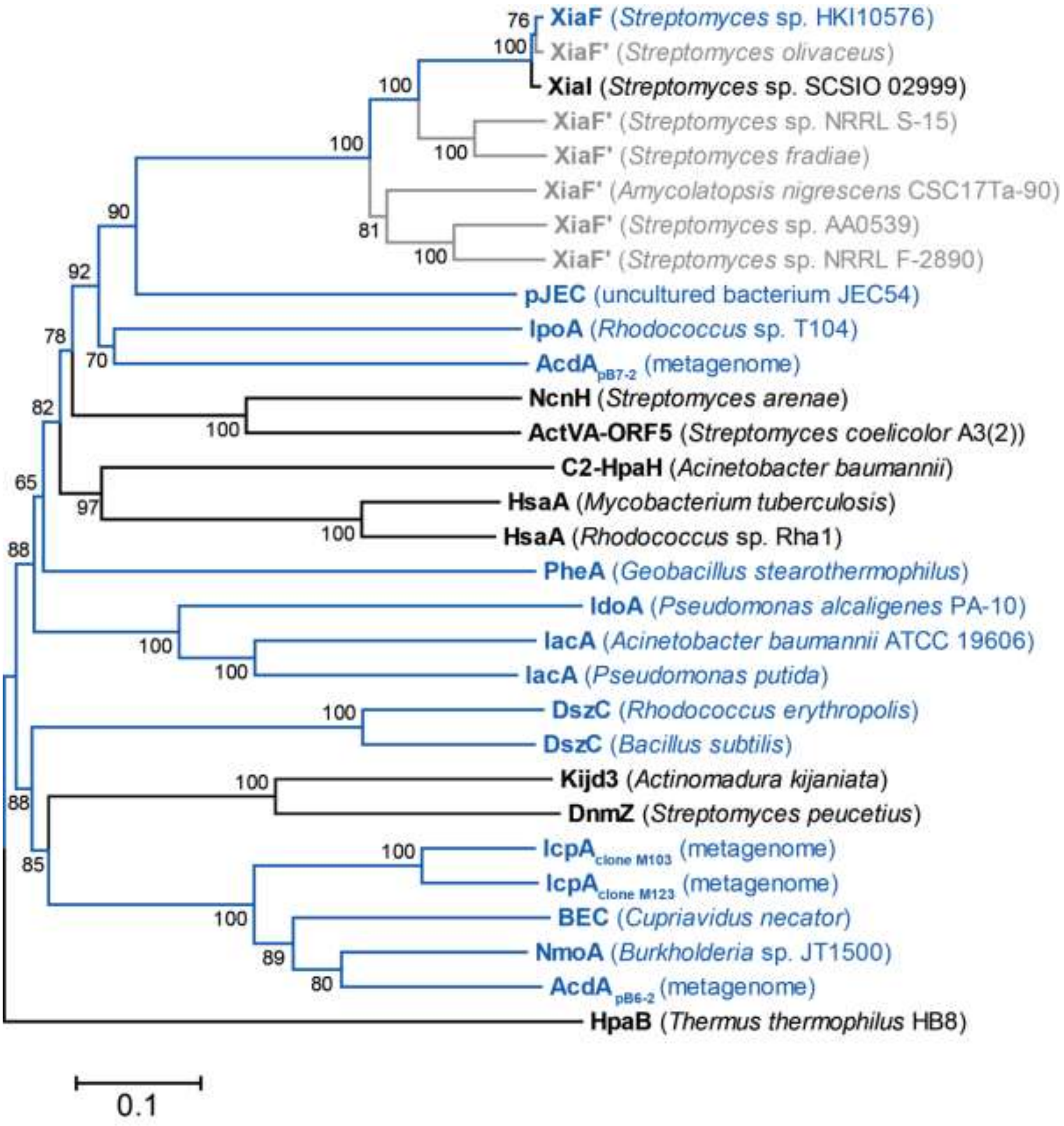

Supplementary Figure 8. Detailed Cladogram of XiaF and Related Flavoenzymes (Neighbor-Joining Method)[49]

The percentage of replicate trees in which the associated taxa clustered together in the bootstrap test (1,000 replicates) is shown next to the branches. Enzymes that have been reported to catalyze indigo formation are colored in blue; deduced gene products (XiaF orthologs) of other putative *xia* gene clusters that have not been characterized are colored in grey.

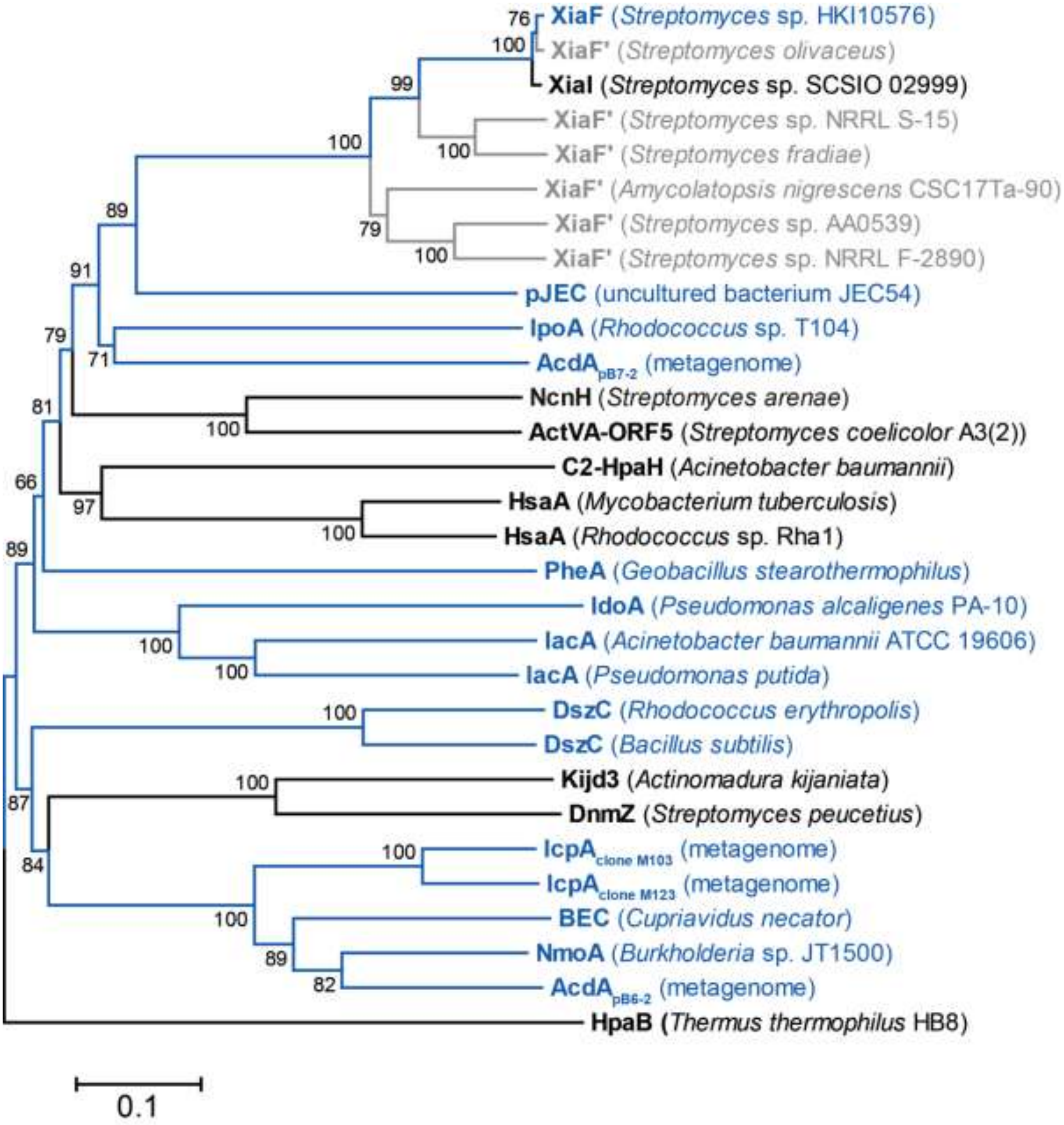

Supplementary Figure 9. Detailed Cladogram of XiaF and Related Flavoenzymes (Minimal Evolution Method)[50]

The percentage of replicate trees in which the associated taxa clustered together in the bootstrap test (1,000 replicates) is shown next to the branches. Enzymes that have been reported to catalyze indigo formation are colored in blue; deduced gene products (XiaF orthologs) of other putative *xia* gene clusters that have not been characterized are colored in grey.

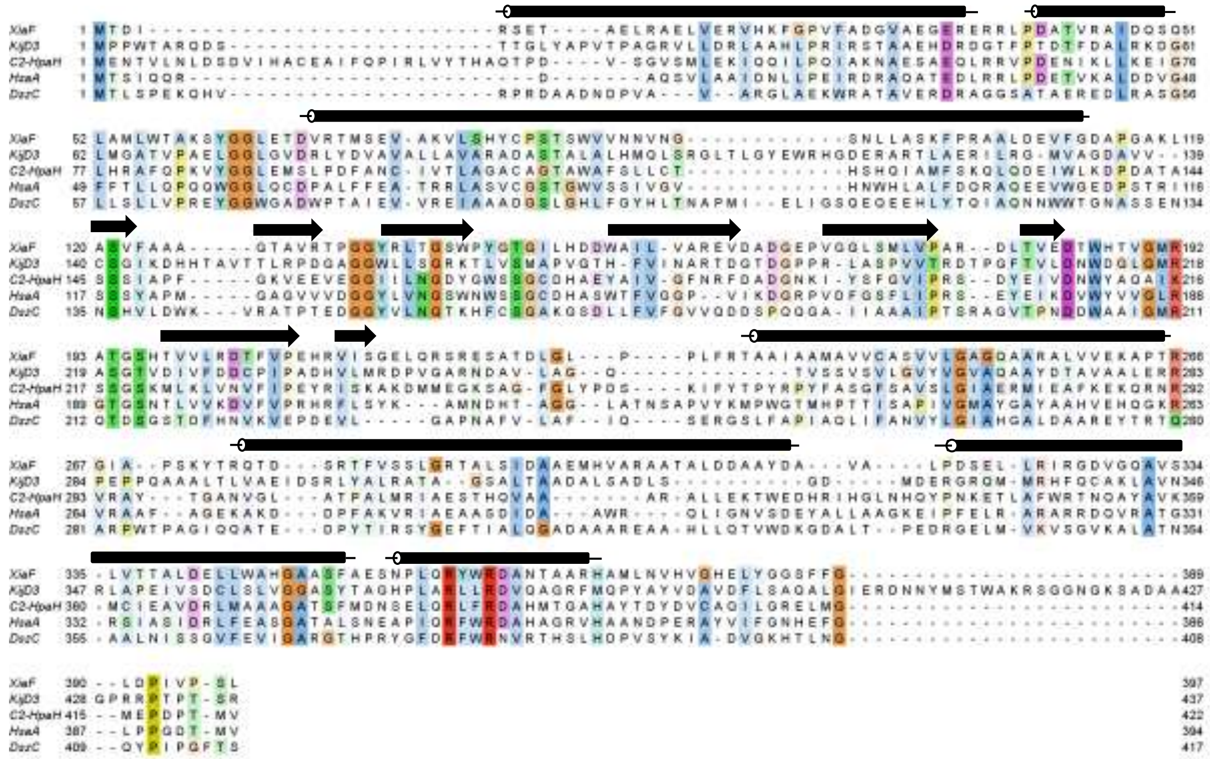

Supplementary Figure 10. Primary Sequence Alignment of XiaF and Crystallized Homologs

Included are the sequences of XiaF, the 4-hydroxyphenylacetate 3-hydroxylase C2-HpaH (PDB ID: 2JBT), the secosteroid hydroxylase HsaA from *Mycobacterium tuberculosis* (PDB ID: 3AFE), the dibenzothiophene monooxygenase DszC from *Rhodococcus erythropolis* D-1 (PDB ID: 3X0Y), and the *N*-oxygenases KijD3 involved in D-kijanose biosynthesis (PDB ID: 4KCF). Residues with high similarity are colored, secondary structure elements are indicated above the sequence (α-helices are indicated in black bars, β-sheets are represented in black arrows).

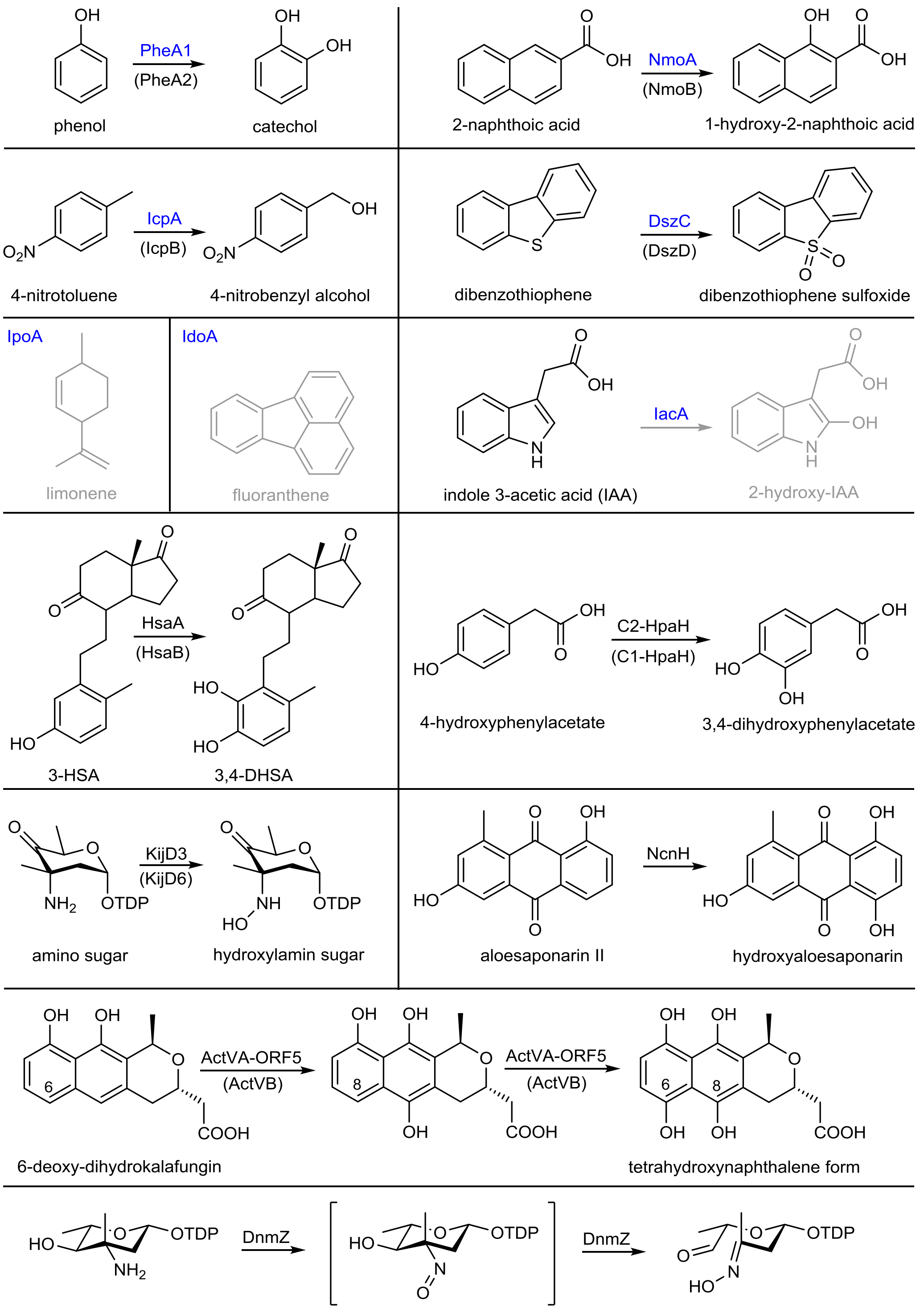

Supplementary Figure 11. Supplementary Information on XiaF Homologs

A detailed overview of confirmed or proposed (grey) substrates, products and enzyme reactions of XiaF homologs (Figure 2B) are displayed. Enzymes that have been shown to catalyze the conversion of indole to indigoid pigments are colored in blue. Proposed flavin reductase partners of homologs are in brackets; 3-HAS: 3-hydroxy-9,10-seconandrost-1,3,5(10)-triene-9,17-dione; 3,9-DHSA: 3,9-dihydroxy-9,10-seconandrost-1,3,5(10)-triene-17-one; TDP: thymidine diphosphate.

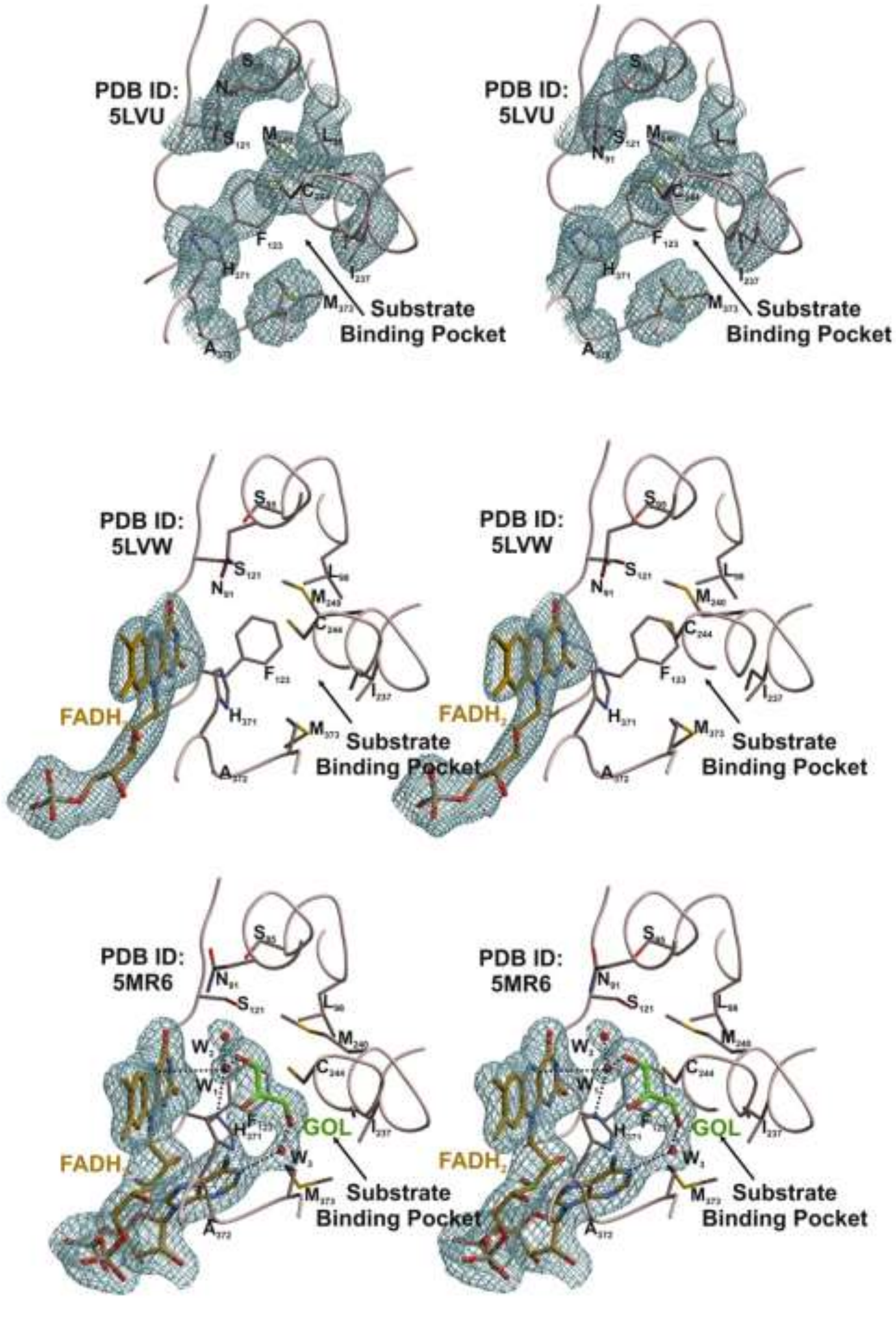

Supplementary Figure 12. Stereo Views and Electron Density Maps

Structures of XiaF (PDB ID: 5LVU), XiaF:FADH 2 (PDB ID: 5LVW) and XiaF:FADH 2:Glycerol (PDB ID: 5MR6) are displayed. Active site residues forming the substrate-binding pocket are labeled according to the protein sequence. Water molecules (W1-3) are shown as red spheres; black dotted lines indicate hydrogen bonds and key interactions. In the XiaF:FADH2:Glycerol complex structure (PDB ID: 5MR6), W1 near the isoalloxazine ring of FADH2 might mimic the binding site of the oxygen substrate. His371 is positioned in close proximity, playing an important role in catalysis by stabilizing the transient peroxide. The 2FO-FC electron density maps (shown in blue) are contoured to 1.0 σ, respectively. All amino acids, ligands and water molecules have been excluded prior to phase calculations to avoid any model bias.

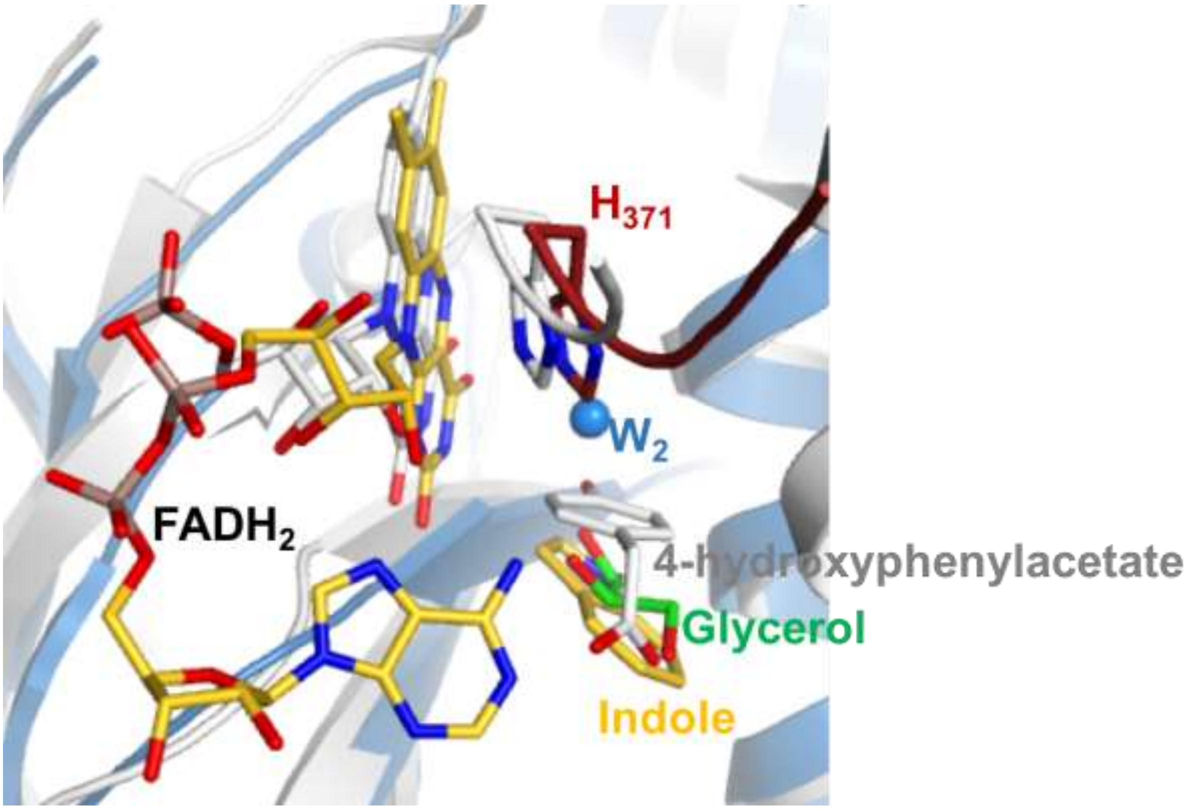

Supplementary Figure 13. Active Site Alignment

Comparison of XiaF and 4-hydroxyphenylacetate 3-hydroxylase. A combinatorial extension alignment[51] of XiaF:$FADH_2$:Glycerol (light blue and firebrick red, PDB ID: 5MR6) and 4-hydroxyphenylacetate 3-hydroxylase (grey, PDB ID: 2JBT) is shown (RMSD 2.77 over 384 residues). The alignment highlights the highly conserved $FADH_2$ coordination. Further, the alignment shows that the glycerol ligand is exactly positioned like the natural ligand 4-hydroxyphenylacetate. The indole ligand, which has been docked with AutoDock Vina[52], exactly occupies this substrate pocket as well. The amino acid is labeled according the one-letter code.

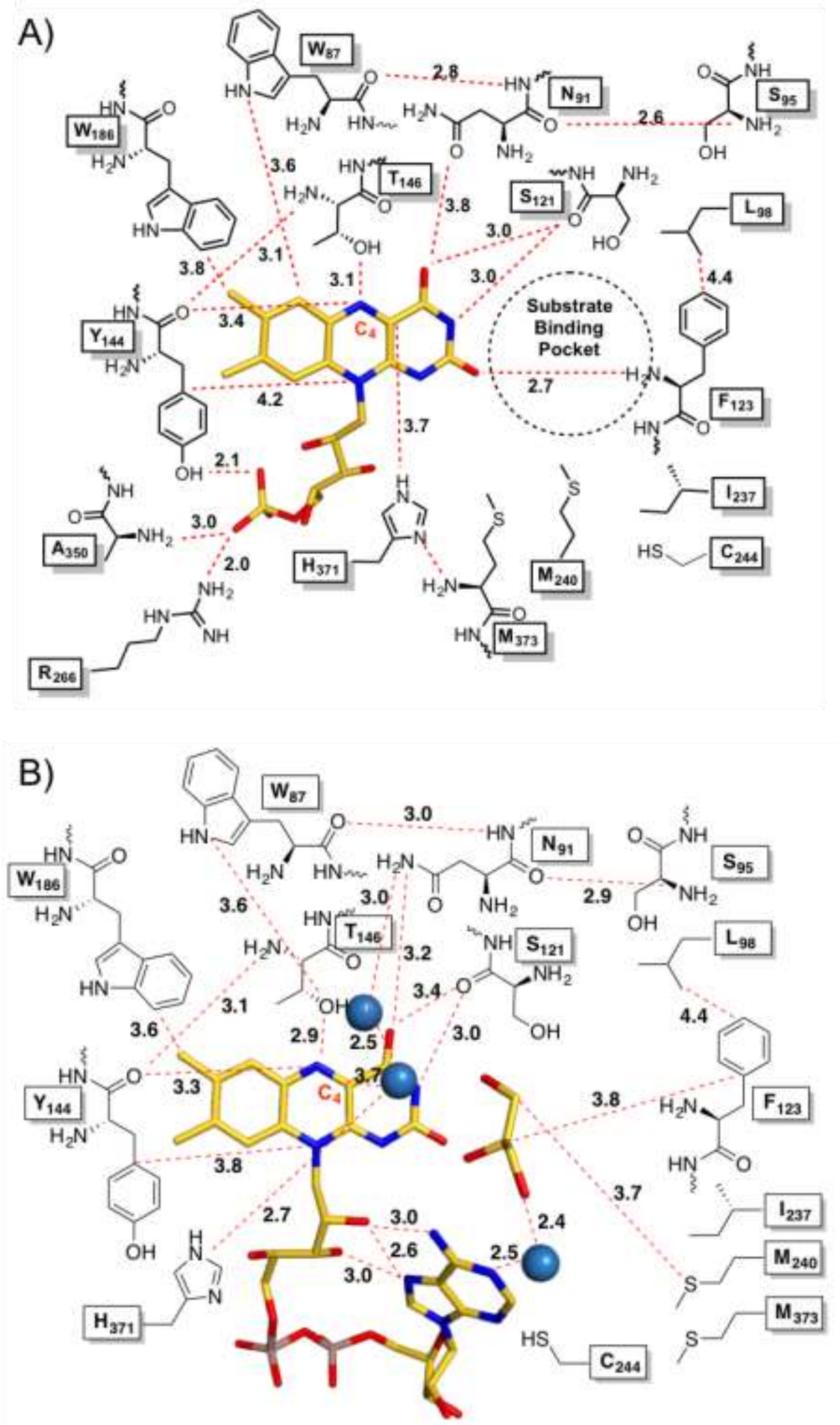

Supplementary Figure 14. Coordination Spheres of XiaF Ligands

A) Coordination sphere of XiaF in complex with $FADH_2$ (PDB ID: 5LVW). $FADH_2$ (colored in gold) is shown in its elongated conformation. B) Coordination sphere of XiaF in complex with $FADH_2$ (colored in gold), glycerol (colored in gold) and H_2O molecules $W_{1\text{-}3}$ (colored in blue, PDB ID: 5MR6). $FADH_2$ is shown in its bent conformation. Amino acids are labeled according to the one-letter code. Red dashed lines indicate closest contact between atoms. Distances are given in Å.

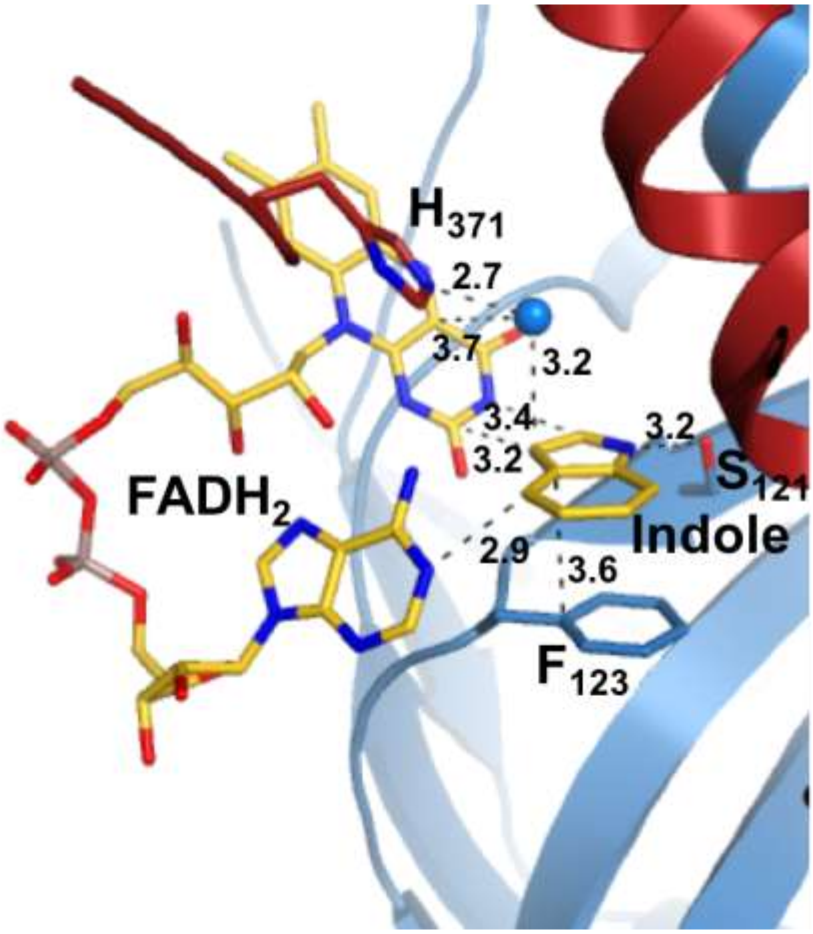

Supplementary Figure 15. Docking of the Indole Ligand into the Active Site of XiaF

Based on the XiaF:$FADH_2$:Glycerol complex structure (PDB ID: 5MR6), indole was docked to the active site using AutoDock Vina[52]. Amino acids are labeled according to the one-letter code. Distances between F_{123}, H_{371} and the $FADH_2$ cosubstrate as well as the indol and water molecules are given in Å.

Supplementary Tables

Supplementary Table 1. Kinetic Parameters of XiaP for FAD, FMN and riboflavin

	substrate 2, constant	K_M^{app} in µM	± s.d. (K_M^{app}) in µM	k_{cat} in min^{-1}	k_{cat}/K_M in min^{-1} x $µM^{-1}$
**FAD**	NADH	9	3	155.2	17.2
**FMN**	NADH	3	1	174.5	58.2
**riboflavin**	NADH	5	3	174.5	34.9

Experiments were performed in 100 µL total volumes with 0.1 µM XiaP and 1.5 mM NADH in assay buffer (20 mM Tris-HCl pH 8.0, 10 % glycerol). Flavin concentrations ranged from 5 to 150 µM. Absorbances at 340 nm were measured in the dark at 28 °C. Initial decrease of the NADH concentration over time was used to calculate initial velocities (v_0). K_M^{app} values were determined by double reciprocal plotting of initial velocities (v_o^{-1}) and substrate concentration ($1/[S]_0$). When applicable, values are shown ± standard deviation (s.d.).

Supplementary Table 2. Kinetic Parameters of XiaP for NADH

	substrate 2, constant	K_M^{app} in µM	± s.d. (K_M^{app}) in µM	k_{cat} in min^{-1}	k_{cat}/K_M in min^{-1} x $µM^{-1}$
**NADH**	FMN	223	27	57	0.3
**NADH**	riboflavin	248	72	52	0.2

Experiments were performed in 120 µL total volumes with 0.1 µM XiaP and 42 µM FMN or riboflavin in assay buffer (20 mM Tris-HCl, pH 8.0, 10 % glycerol). NADH concentrations ranged from 4.1 to 2,500 µM for FMN and from 12.5 to 2,500 µM for riboflavin. Absorbances at 340 nm were measured in the dark at 28 °C. Initial decrease of the NADH concentration over time was used to calculate initial velocities (v_0). K_M^{app} values were determined by double reciprocal plotting of initial velocities (v_o^{-1}) and substrate concentration ($[S]_0^{-1}$). When applicable, values are shown ± standard deviation (s.d.).

Supplementary Table 3. Data Collection and Refinement Statistics of Crystallization Experiments

	**XiaF: apo**	**XiaF: $FADH_2$**	**XiaF: $FADH_2$ + Glycerol**
Space group	$P6_222$	$P6_222$	P1
Cell constants (Å)	a = b = 166.5<br>c = 99.0	a = b = 166.6<br>c = 99.7	a = 166.9<br>b = 167.1<br>c = 200.9<br>α = 90.0<br>β = 90.2<br>γ = 119.8
Molecules in asym. unit	1	1	24
Disordered regions	1-4	1-4	-
X-ray source	SLS, X06SA	SLS, X06SA	SLS, X06SA
Wavelength (Å)	1.0	1.0	1.0
Resolution range (Å)[a]	50-2.6 (2.7-2.6)	50-2.9 (3.0-2.9)	50-2.4 (2.5-2.4)
No. observations	275883	162141	1271109
No. unique reflections	25318[b]	17733	703582
Completeness (%)[b]	99.6 (99.7)	90.3 (96.9)	95.5 (94.8)
R_{merge} (%)[a,b]	8.5 (56.3)	8.4 (52.5)	9.8 (57.8)
CC1/2	94.5	95.8	65.9
I/σ (I)[a]	16.5 (4.3)	18.8 (5.9)	7.4 (1.8)
Resolution range (Å)	15-2.6	15-2.9	15-2.4
No. reflections working set	24035	16846	665721
No. reflections test set	1265	887	35038
No. non hydrogen (protein)	2954	2954	77543
No. heteroatoms $FADH_2$, GOL	-	31	1416
H_2O. ions	72	30	4679
R_{work}/R_{free} (%)[c]	18.2 / 21.8	17.4 / 20.2	19.8 / 22.9
r.m.s.d. bond lengths (Å) / (°)[d]	0.004 / 0.788	0.004 / 0.846	0.012 / 1.654
Average B-factor ($Å^2$)			
Protein	65	66	39
Ligand ($FADH_2$; GOL)	-	90	44
Solvent (water; ions)	66	66	40
Ramachandran Plot (%)[e]	98.5 / 1.5 / 0.0	98.7 / 1.3 / 0.0	99.0 / 1.0 / 0.0
PDB accession code	5LVU	5LVW	5MR6

[a] The values in parentheses of resolution range, completeness, R_{merge} and I/σ (I) correspond to the last resolution shell. [b] $R_{merge}(I) = \sum_{hkl}\sum_j |[I(hkl)_j - I(hkl)]| / \sum_{hkl} I_{hkl}$, where $I(hkl)_j$ is the measurement of the intensity of reflection *hkl* and <I(*hkl*)> is the average intensity. [c] $R = \sum_{hkl} | |F_{obs}| - |F_{calc}| | / \sum_{hkl} |F_{obs}|$, where R_{free} is calculated without a sigma cut off for a randomly chosen 5% of reflections, which were not used for structure refinement, and R_{work} is calculated for the remaining reflections. [d] Deviations from ideal bond lengths/angles. [e] Number of residues in favored region / allowed region / outlier region.

Supplementary References

1 Deng, D., Li, X., Fang, X. & Sun, G. Characterization of two components of the 2-naphthoate monooxygenase system from *Burkholderia* sp. strain JT1500. *FEMS Microbiol. Lett.* **273**, 22-27, (2007).

2 Furuya, T., Takahashi, S., Ishii, Y., Kino, K. & Kirimura, K. Cloning of a gene encoding flavin reductase coupling with dibenzothiophene monooxygenase through coexpression screening using indigo production as selective indication. *Biochem. Biophys. Res. Commun.* **313**, 570-575, (2004).

3 Dresen, C. *et al.* A flavin-dependent monooxygenase from *Mycobacterium tuberculosis* involved in cholesterol catabolism. *J. Biol. Chem.* **285**, 22264-22275, (2010).

4 Van der Geize, R. *et al.* A gene cluster encoding cholesterol catabolism in a soil actinomycete provides insight into *Mycobacterium tuberculosis* survival in macrophages. *Proc. Natl. Acad. Sci. U.S.A.* **104**, 1947-1952, (2007).

5 Thotsaporn, K., Sucharitakul, J., Wongratana, J., Suadee, C. & Chaiyen, P. Cloning and expression of *p*-hydroxyphenylacetate 3-hydroxylase from *Acinetobacter baumannii*: evidence of the divergence of enzymes in the class of two-protein component aromatic hydroxylases. *Biochim. Biophys. Acta* **1680**, 60-66, (2004).

6 Valton, J., Filisetti, L., Fontecave, M. & Niviere, V. A two-component flavin-dependent monooxygenase involved in actinorhodin biosynthesis in *Streptomyces coelicolor*. *J. Biol. Chem.* **279**, 44362-44369, (2004).

7 Al-Mestarihi, A., Romo, A., Liu, H. W. & Bachmann, B. O. Nitrososynthase-triggered oxidative carbon−carbon bond cleavage in baumycin biosynthesis. *J. Am. Chem. Soc.* **135**, 11457-11460, (2013).

8 Zeng, J. & Zhan, J. A novel fungal flavin-dependent halogenase for natural product biosynthesis. *ChemBioChem* **11**, 2119-2123, (2010).

9 Shepherd, S. A. *et al.* Extending the biocatalytic scope of regiocomplementary flavin-dependent halogenase enzymes. *Chem. Sci.* **6**, 3454-3460, (2015).

10 Van Lanen, S. G., Lin, S., Horsman, G. P. & Shen, B. Characterization of SgcE6, the flavin reductase component supporting FAD-dependent halogenation and hydroxylation in the biosynthesis of the enediyne antitumor antibiotic C-1027. *FEMS Microbiol. Lett.* **300**, 237-241, (2009).

11 Gaudu, P., Touati, D., Niviere, V. & Fontecave, M. The NAD(P)H:flavin oxidoreductase from *Escherichia col*i as a source of superoxide radicals. *J. Biol. Chem.* **269**, 8182-8188, (1994).

12 Li, H. *et al.* Elucidating the cyclization cascades in xiamycin biosynthesis by substrate synthesis and enzyme characterizations. *Org. Lett.* **17**, 306-309, (2015).

13 Li, H. *et al.* Identification and characterization of xiamycin A and oxiamycin gene cluster reveals an oxidative cyclization strategy tailoring indolosesquiterpene biosynthesis. *J. Am. Chem. Soc.* **134**, 8996-9005, (2012).

14 Huijbers, M. M., Montersino, S., Westphal, A. H., Tischler, D. & van Berkel, W. J. Flavin dependent monooxygenases. *Arch. Biochem. Biophys.* **544**, 2-17, (2014).

15 Lim, H. K. *et al.* Characterization of a forest soil metagenome clone that confers indirubin and indigo production on *Escherichia coli*. *Appl. Environ. Microbiol.* **71**, 7768-7777, (2005).

16 Kim, I. C. & Oriel, P. J. Characterization of the *Bacillus stearothermophilus* BR219 phenol hydroxylase gene. *Appl. Environ. Microbiol.* **61**, 1252-1256, (1995).

17 Drewlo, S., Bramer, C. O., Madkour, M., Mayer, F. & Steinbuchel, A. Cloning and expression of a *Ralstonia eutropha* HF39 gene mediating indigo formation in *Escherichia coli*. *Appl. Environ. Microbiol.* **67**, 1964-1969, (2001).

18 Alemayehu, D., Gordon, L. M., O'Mahony, M. M., O'Leary, N. D. & Dobson, A. D. Cloning and functional analysis by gene disruption of a novel gene involved in indigo production and

fluoranthene metabolism in *Pseudomonas alcaligenes* PA-10. *FEMS Microbiol. Lett.* **239**, 285-293, (2004).
19 Leveau, J. H. & Gerards, S. Discovery of a bacterial gene cluster for catabolism of the plant hormone indole 3-acetic acid. *FEMS Microbiol. Ecol.* **65**, 238-250, (2008).
20 Coco, W. M. *et al.* DNA shuffling method for generating highly recombined genes and evolved enzymes. *Nat. Biotechnol.* **19**, 354-359, (2001).
21 Lin, G. H. *et al.* Identification and characterization of an indigo-producing oxygenase involved in indole 3-acetic acid utilization by *Acinetobacter baumannii*. *Antonie Van Leeuwenhoek* **101**, 881-890, (2012).
22 Hart, S., Koch, K. R. & Woods, D. R. A gene cluster encoding cholesterol catabolism in a soil actinomycete provides insight into *Mycobacterium tuberculosis* survival in macrophages. *J. Gen. Microbiol.* **138**, 211-216, (1992).
23 Kimura, N., Sakai, K. & Nakamura, K. Isolation and characterization of a 4-nitrotoluene-oxidizing enzyme from activated sludge by a metagenomic approach. *Microbes Environ.* **25**, 133-139, (2010).
24 Nagayama, H. *et al.* Isolation of oxygenase genes for indigo-forming activity from an artificially polluted soil metagenome by functional screening using *Pseudomonas putida* strains as hosts. *Appl. Microbiol. Biotechnol.* **99**, 4453-4470, (2015).
25 Choi, K. Y. *et al.* Molecular cloning and identification of a novel oxygenase gene specifically induced during the growth of *Rhodococcus* sp. Strain T104 on limonene. *J. Microbiol.* **42**, 160-162, (2004).
26 Lee, J., Jayaraman, A. & Wood, T. K. Indole is an inter-species biofilm signal mediated by SdiA. *BMC Microbiol.* **7**, 42, (2007).
27 Lee, J. H. & Lee, J. Indole as an intercellular signal in microbial communities. *FEMS Microbiol. Rev.* **34**, 426-444, (2010).
28 Lin, G. H., Chen, H. P. & Shu, H. Y. Detoxification of indole by an indole-induced flavoprotein oxygenase from *Acinetobacter baumannii*. *PLoS ONE* **10**, e0138798, (2015).
29 Fernández-Arrojo, L., Guazzaroni, M.-E., López-Cortés, N., Beloqui, A. & Ferrer, M. Metagenomic era for biocatalyst identification. *Curr. Opin. Biotechnol.* **21**, 725-733, (2010).
30 Taguchi, T. *et al.* Biosynthetic conclusions from the functional dissection of oxygenases for biosynthesis of actinorhodin and related *Streptomyces* antibiotics. *Chem. Biol.* **20**, 510-520, (2013).
31 Brunker, P., Sterner, O., Bailey, J. E. & Minas, W. Heterologous expression of the naphthocyclinone hydroxylase gene from *Streptomyces arenae* for production of novel hybrid polyketides. *Antonie Leeuwenhoek* **79**, 235-245, (2001).
32 Thoden, J. B., Branch, M. C., Zimmer, A. L., Bruender, N. A. & Holden, H. M. Active site architecture of a sugar *N*-oxygenase. *Biochemistry* **52**, 3191-3193, (2013).
33 Omokoko, B., Jantges, U. K., Zimmermann, M., Reiss, M. & Hartmeier, W. Isolation of the *phe*-operon from *G. stearothermophilus* comprising the phenol degradative *meta*-pathway genes and a novel transcriptional regulator. *BMC Microbiol.* **8**, 197, (2008).
34 Xi, L., Squires, C. H., Monticello, D. J. & Childs, J. D. A flavin reductase stimulates DszA and DszC proteins of *Rhodococcus erythropolis* IGTS8 *in vitro*. *Biochem. Biophys. Res. Commun.* **230**, 73-75, (1997).
35 Kamali, N., Tavallaie, M., Bambai, B., Karkhane, A. A. & Miri, M. Site-directed mutagenesis enhances the activity of NADH-FMN oxidoreductase (DszD) activity of *Rhodococcus erythropolis*. *Biotechnol. Lett.* **32**, 921-927, (2010).
36 Kirimura, K. *et al.* Identification and functional analysis of the genes encoding dibenzothiophene-desulfurizing enzymes from thermophilic bacteria. *Appl. Microbiol. Biotechnol.* **65**, 703-713, (2004).
37 Scott, J. C., Greenhut, I. V. & Leveau, J. H. J. Functional characterization of the bacterial *iac* genes for degradation of the plant hormone indole-3-acetic acid. *J. Chem. Ecol.* **39**, 942-951, (2013).

38 Alfieri, A. *et al.* Structure of the monooxygenase component of a two-component flavoprotein monooxygenase. *Proc. Natl. Acad. Sci. U.S.A.* **104**, 1177-1182, (2007).
39 Chaiyen, P., Suadee, C. & Wilairat, P. A novel two-protein component flavoprotein hydroxylase – *p*-hydroxyphenylacetate hydroxylase from *Acinetobacter baumannii*. *Eur. J. Biochem.* **268**, 5550-5561, (2001).
40 Bruender, N. A., Thoden, J. B. & Holden, H. M. X-ray structure of KijD3, a key enzyme involved in the biosynthesis of D-kijanose. *Biochemistry* **49**, 3517-3524, (2010).
41 Zhang, H. *et al.* Elucidation of the kijanimicin gene cluster: insights into the biosynthesis of spirotetronate antibiotics and nitrosugars. *J. Am. Chem. Soc.* **129**, 14670-14683, (2007).
42 Sartor, L., Ibarra, C., Al-Mestarihi, A., Bachmann, B. O. & Vey, J. L. Structure of DnmZ, a nitrososynthase in the *Streptomyces peucetius* anthracycline biosynthetic pathway. *Acta Crystallogr. F* **71**, 1205-1214, (2015).
43 Okamoto, S., Taguchi, T., Ochi, K. & Ichinose, K. Biosynthesis of actinorhodin and related antibiotics: discovery of alternative routes for quinone formation encoded in the *act* gene cluster. *Chem. Biol.* **16**, 226-236, (2009).
44 Gray, K. A. *et al.* Molecular mechanisms of biocatalytic desulfurization of fossil fuels. *Nat. Biotechnol.* **14**, 1705-1709, (1996).
45 Sievers, F. *et al.* Fast, scalable generation of high-quality protein multiple sequence alignments using Clustal Omega. *Mol. Syst. Biol.* **7**, 539, (2011).
46 Yang, J. *et al.* The I-TASSER Suite: protein structure and function prediction. *Nat. Methods* **12**, 7-8, (2015).
47 Bak, S. *et al.* Cyanogenic glycosides: a case study for evolution and application of cytochromes P450. *Phytochem. Rev.* **5**, 309-329, (2006).
48 Holm, L. & Rosenstrom, P. Dali server: conservation mapping in 3D. *Nucleic Acids Res.* **38**, W545-549, (2010).
49 Saitou, N. & Nei, M. The neighbor-joining method: a new method for reconstructing phylogenetic trees. *Mol. Biol. Evol.* **4**, 406-425, (1987).
50 Rzhetsky, A. & Nei, M. A simple method for estimating and testing minimum-evolution trees. *Mol. Biol. Evol.* **9**, 945-967, (1992).
51 Shindyalov, I. N. & Bourne, P. E. Protein structure alignment by incremental combinatorial extension (CE) of the optimal path. *Protein. Eng.* **11**, 739-747, (1998).
52 Trott, O. & Olson, A. J. AutoDock Vina: improving the speed and accuracy of docking with a new scoring function, efficient optimization, and multithreading. *J. Comput. Chem.* **31**, 455–461, (2010).
